# An epidemic Zika virus isolate suppresses antiviral immunity by disrupting antigen presentation pathways

**DOI:** 10.1038/s41467-021-24340-0

**Published:** 2021-06-30

**Authors:** Ryan D. Pardy, Stefanie F. Valbon, Brendan Cordeiro, Connie M. Krawczyk, Martin J. Richer

**Affiliations:** 1 Department of Microbiology & Immunology, McGill University, Montreal, QC Canada; 2 Rosalind & Morris Goodman Cancer Research Centre, McGill University, Montreal, QC Canada; 3 Department of Metabolism and Nutritional Programming, Van Andel Research Institute, Grand Rapids, MI USA; 4 Present Address: Department of Microbiology and Immunology, Indiana University School of Medicine, Indianapolis, IN USA

**Keywords:** Antigen presentation, Lymphocyte activation, Viral infection, Viral immune evasion

## Abstract

Zika virus (ZIKV) has emerged as an important global health threat, with the recently acquired capacity to cause severe neurological symptoms and to persist within host tissues. We previously demonstrated that an early Asian lineage ZIKV isolate induces a highly activated CD8 T cell response specific for an immunodominant epitope in the ZIKV envelope protein in wild-type mice. Here we show that a contemporary ZIKV isolate from the Brazilian outbreak severely limits CD8 T cell immunity in mice and blocks generation of the immunodominant CD8 T cell response. This is associated with a more sustained infection that is cleared between 7- and 14-days post-infection. Mechanistically, we demonstrate that infection with the Brazilian ZIKV isolate reduces the cross-presentation capacity of dendritic cells and fails to fully activate the immunoproteasome. Thus, our study provides an isolate-specific mechanism of host immune evasion by one Brazilian ZIKV isolate, which differs from the early Asian lineage isolate and provides potential insight into viral persistence associated with recent ZIKV outbreaks.

## Abstract (web summary)

The CD8 T cell response to Zika virus is known to be a critical component of the host immune response to infection. Here the authors show a Zika virus isolate specific disruption of antigen processing that impacts the host response and impairs viral clearance providing evidence of isolate specific impacts on the immune response to infection

## Introduction

Zika virus (ZIKV) is a mosquito-borne pathogen of the *Flaviviridae* family (*Flavivirus* genus), which was first isolated in the Zika Forest region of Uganda in 1947^1^. From this initial isolation through the remainder of the twentieth century, only a small number of isolated infections were reported^2,3^. However, in April–July 2007 ZIKV was identified as the cause of a major outbreak on Yap Island, Federated States of Micronesia, infecting approximately 73% of the island’s population^4^. This was followed by another major outbreak in French Polynesia, during which ~32,000 people were infected over the course of 6 months in 2013^5^. More recently, ZIKV caused a major epidemic that began in Brazil in 2015, before spreading rapidly throughout South and Central America, with some local transmission in the southern United States^3,6^. During this epidemic, ZIKV infections were reported in 84 countries worldwide, and over 800,000 suspected and confirmed cases were reported in the Americas alone^6^. Together, these outbreaks represent a striking change in phenotype for ZIKV and suggest that this previously innocuous virus may have acquired a novel epidemic capacity.

The French Polynesian and South and Central American outbreaks also marked the first instances in which ZIKV infection was associated with severe neurological symptoms. ZIKV infection during pregnancy can cause fetal microcephaly, while the most common symptom observed in adults is Guillain–Barré Syndrome, an autoimmune ascending paralysis^7–11^. However other neurological symptoms, as well as uveitis and hematospermia (blood in the semen) have also been reported in adults, and correlate with the detection of ZIKV RNA in cerebrospinal fluid, aqueous humor, and semen, respectively^11^. It has also been reported that ZIKV RNA may be persistently detected in some tissues. In women, ZIKV RNA may be detected in vaginal secretions until 13 days post-onset of symptoms (POS), while in men, some semen samples remained positive over 100 days POS^11–13^. Likewise, in both men and women ZIKV RNA has been detected as late as 36 days POS in urine and 15 days POS in serum^11,12,14^. Together, the apparent increase in epidemic capacity, novel association with neurological symptoms, and persistence of ZIKV RNA suggest that recent ZIKV isolates may have evolved to counter host immunity and establish more sustained infections.

Several studies have begun to compare epidemic and historical ZIKV isolates in order to understand whether contemporary isolates have an enhanced capacity to evade host immunity. Genetic analyses of ZIKV have identified two main lineages, African and Asian^15^. All of the outbreaks described above were caused by Asian lineage isolates, some of which have been found to possess conserved mutations that correlate with neuroinvasion, evasion of the type I interferon (IFN-I) response, and microcephaly^16–18^. Comparisons of African and Asian lineage ZIKV infection in A129 mice, which lack the IFN-α/β receptor (IFNAR), and STAT2 KO mice have found that infection with African lineage ZIKV was lethal, while mice survived infection with a Puerto Rican ZIKV isolate (Asian lineage)^19,20^. In STAT2 KO mice, mortality correlated with induction of IFN-α and IFN-β mRNA, with the highest levels observed following infection with the Ugandan isolate MR766 (African lineage)^19^. Although these studies were undertaken in immunocompromised hosts, which limits their usefulness in interrogating immune responses to infection, they suggest that contemporary ZIKV isolates induce a less inflammatory and less lethal infection than historical ZIKV isolates. However, it is unknown whether some epidemic isolates of ZIKV have improved capacity to evade host immunity compared to earlier, pre-epidemic Asian lineage isolates.

Improving our understanding of the immune response to ZIKV, and how this relates to sustained virus detection and the neurological symptoms associated with infection is a priority for addressing this major public health concern. Although no outbreaks are currently ongoing, 90 countries and territories remain at risk of ZIKV transmission (based on past local transmission), and another 56 have the mosquito vector but have not yet reported transmission^21^. To address whether the immune response to ZIKV has changed, our group previously established an immunocompetent mouse model of ZIKV infection^22^. Our data demonstrated that an early Asian lineage ZIKV isolate (PLCal_ZV, accession number KF993678; referred to herein as ZIKV^CDN^) establishes an active, albeit rapidly cleared, infection in C57BL/6 wild type (WT) mice^22^. This leads to a robust and prototypical antiviral innate immune response, including dendritic cell (DC) and natural killer cell activation^22^. Furthermore, we have used a previously described surrogate marker approach to identify and track the total antigen-experienced CD8 T cell response to ZIKV infection^22,23^. Importantly, this approach enabled us to identify an immunodominant CD8 T cell epitope in the ZIKV envelope protein (Env_294–302_), which has also been independently described by other groups^22,24,25^. Thus, our model provides an important baseline and an opportunity for comparisons of the immune response to historical and contemporary ZIKV isolates. In addition, since the mice are immunocompetent and do not succumb to infection, this model allows us to query the impact of epidemic ZIKV isolates on antiviral cytotoxic T cell responses.

Herein, we demonstrate that a Brazilian ZIKV isolate (HS-2015-BA-01, accession number KX520666; referred to herein as ZIKV^BR^) actively suppresses CD8 T cell immunity following infection. This correlates with reduced IFN-I production, reduced activation of DCs, inhibition of cross-presentation, and failure to induce immunoproteasome activation, which results in the complete abrogation of the immunodominant Env_294–302_-specific CD8 T cell response. Together, these factors result in sustained, but not chronic, infection with replicative virus detected as late as 7 days following infection. Thus, our findings suggest that this epidemic ZIKV isolate has developed a number of ways to either avoid or actively counter immune responses. Although the current study is limited to one epidemic isolate, our data provide some insight that could potentially explain at least one of the mechanisms behind the increased pathogenesis and a broader scope of transmission associated with ZIKV infection during the 2015 outbreak in the Americas.

## Results

### Reduced CD8 T cell response to ZIKV^BR^ infection correlates with virus detection at later time points post-infection

We previously characterized the T cell response to an early Asian lineage ZIKV isolate PLCal_ZV (accession number KF993678; referred to herein as ZIKV^CDN^) in immunocompetent mice^22,26^. This isolate was taken from a Canadian patient who acquired the infection in Thailand in 2013 (prior to the South and Central American outbreak), sequenced and determined to belong to the Asian lineage, and was reported to be associated with mild symptoms^26^. To determine whether an epidemic ZIKV isolate differs in its capacity to induce CD8 T cell responses, we compared the response induced by infection with ZIKV^CDN^ to the response induced by infection with HS-2015-BA-01 (accession number KX520666; referred to herein as ZIKV^BR^), a Brazilian isolate from the 2015 South and Central American outbreak. This isolate was obtained from a viremic patient in the Bahia state of Brazil, fully sequenced, and has been characterized in both cell culture and in animal models^27–32^. Although both isolates fall within the Asian lineage, there are 13 amino acid polymorphisms between these two ZIKV isolates, five of which are located in the non-structural (NS)5 protein, two each in the premembrane (PrM), NS3, and NS4A proteins, and one each in the capsid and NS2A proteins^27^. These differences have been demonstrated to lead to different phenotypes between these two viruses in cell culture models, with ZIKV^BR^ causing more cytopathic effects and increased replicative efficiency in certain cell types^27,28^. Despite these studies, whether these isolates induce different immune responses in immunocompetent mice remained unknown.

**Fig. 1:**
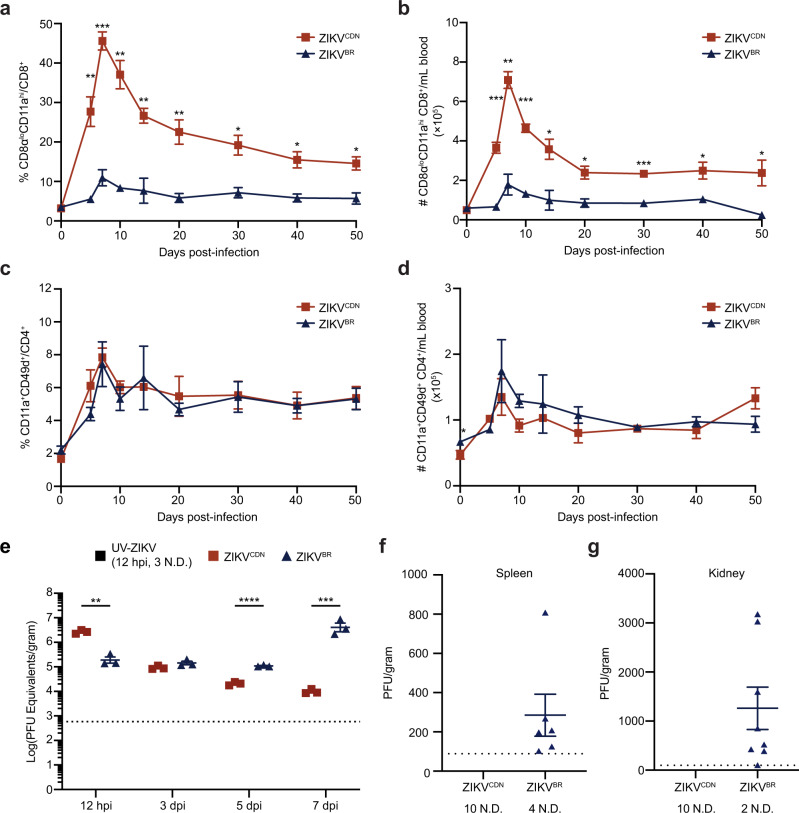
Reduced CD8 T cell response to ZIKV^BR^ is associated with virus detection at later time points following infection. **a**, **b** Frequency (**a**) and number (**b**) of CD8α^lo^CD11a^hi^ CD8 T cells in the peripheral blood of mice at indicated dpi with ZIKV^CDN^ or ZIKV^BR^. Data are representative of two independent experiments with *n* = 3 mice per group, sampled repeatedly at indicated time points. Data were analyzed by two-tailed, unpaired Student’s *t*-test at each time point. In **a** day 5 *p* = 0.0041, day 7 *p* = 0.0004, day 10 *p* = 0.0015, day 14 *p* = 0.0067, day 20 *p* = 0.0073, day 30 *p* = 0.0132, day 40 *p* = 0.0137, day 50 *p* = 0.0157. In **b** day 5 *p* = 0.0004, day 7 *p* = 0.0014, day 10 *p* = 0.0003, day 14 *p* = 0.0218, day 20 *p* = 0.0169, day 30 *p* = 0.0002, day 40 *p* = 0.0371, day 50 *p* = 0.0327. **c**, **d** Frequency (**c**) and number (**d**) of CD11a^+^CD49d^+^ CD4 T cells in the peripheral blood of mice at indicated dpi with ZIKV^CDN^ or ZIKV^BR^. Data are representative of two independent experiments with *n* = 3 mice per group, sampled repeatedly at indicated time points. Data were analyzed by two-tailed, unpaired Student’s *t*-test at each time point. In **d** day 0 *p* = 0.0407. **e** Viral RNA was quantified in the spleen using RT-qPCR analysis. Data are presented as PFU equivalents per gram of tissue after comparison to a standard curve of *C*_t_ value versus log_10_(PFU) for each isolate. Mice were sacrificed 12 hpi and 3, 5, and 7 dpi with ZIKV^CDN^ or ZIKV^BR^, or following mock-infection or injection with UV-ZIKV (12 hpi only). Data are representative of three independent experiments, with *n* = 3 mice per group at each time point. Data were analyzed by two-tailed, unpaired Student’s *t*-test at each time point, 12 hpi *p* = 0.001, 5 dpi *p* = 0.000079, 7 dpi *p* = 0.00014. **f**, **g** Viral burden in the spleen (**f**) and kidney (**g**) were quantified via plaque assay 7 dpi with each ZIKV isolate. N.D. indicates no data recorded above the LOD (dotted line). Data are pooled from two experiments with *n* = 5 mice per group. All data are shown as mean ± SEM and *p < 0.05, **p < 0.01, ***p < 0.001, and ****p < 0.0001. Red squares = ZIKV^CDN^-infected mice, blue triangles = ZIKV^BR^-infected mice, black squares UV-ZIKV-injected mice. Source data are provided as a Source Data file.

We first assessed the total CD8 T cell response to infection with each ZIKV isolate in the blood over time using a surrogate marker approach we have previously validated in the context of ZIKV infection^22^. WT C57BL/6 mice were infected with ZIKV^CDN^ or ZIKV^BR^ and the total antigen-experienced (CD8α^lo^CD11a^hi^) CD8 T cell response was analyzed in the blood at various days post-infection (dpi). We observed that, as previously described^22^, ZIKV^CDN^ induced a robust CD8α^lo^CD11a^hi^ CD8 T cell response that peaked at 7 dpi (Fig. 1a, b). However, both the frequency and number of CD8α^lo^CD11a^hi^ CD8 T cells responding to ZIKV^BR^ infection were severely reduced compared to infection with ZIKV^CDN^ (Fig. 1a, b). In contrast, the CD11a^+^CD49d^+^ (also validated as markers of antigen-experienced CD4 T cells during ZIKV infection^22^) CD4 T cell response was comparable between the two infections (Fig. 1c, d). Thus, our data suggest that ZIKV^BR^ infection suppresses CD8 T cell immunity despite inducing a numerically equivalent CD4 T cell response in the blood.

Given the importance of CD8 T cells for viral clearance, we asked whether the reduced CD8 T cell response to ZIKV^BR^ had an impact on the capacity of the host to control ZIKV infection. To address this question, we quantified ZIKV RNA in the spleen at various time points following infection with ZIKV^CDN^ or ZIKV^BR^. Spleens were harvested at 12 h post-infection (hpi), a time point at which viral load peaks following ZIKV^CDN^ infection^22^, as well as 3, 5, and 7 dpi, to analyze viral kinetics. As additional controls, mice were mock-infected or injected with an equivalent dose of UV-inactivated ZIKV^CDN^ (UV-ZIKV; 12 hpi only). The viral burden was assessed using RT-qPCR and expressed as PFU equivalents per gram of tissue. As previously described^22^, we did not detect any ZIKV RNA above the limit of detection (LOD) in mice that were injected with UV-ZIKV, confirming that infection with live ZIKV is required for detection of viral RNA (Fig. 1e). Although viral load was significantly lower in the spleen 12 hpi with ZIKV^BR^ compared to ZIKV^CDN^, viral load declined 3 dpi with ZIKV^CDN^, while remaining unchanged following ZIKV^BR^ infection (Fig. 1e). Strikingly, and in contrast to declining viral load in ZIKV^CDN^-infected mice during the time course, we observed increasing viral loads at 5 and 7 dpi with ZIKV^BR^ (Fig. 1e). Thus, our data suggest that dampened CD8 T cell responses to ZIKV^BR^ are associated with more sustained detection of viral RNA in WT mice.

In order to confirm that the viral RNA we detected at this late time point was representative of the replicative virus, and to determine whether this led to increased viral dissemination, we measured the amount of infectious virus in the spleen and kidney by plaque assay 7 dpi with each isolate. We detected no ZIKV^CDN^ above the LOD in either organ (Fig. 1f, g). However, infectious ZIKV^BR^ was detectable in 9/10 mice 7 dpi: 5/10 in both the spleen and kidney, 3/10 in the kidney alone, and 1/10 in the spleen alone (Fig. 1f, g). Although the number of PFU per gram of tissue is lower than the number of PFU equivalents per gram of tissue calculated by RT-qPCR (Fig. 1e), this likely reflects that more copies of the genome are produced than infectious virus particles, as well as the potentially higher sensitivity of RT-qPCR. Since the South and Central American epidemic was associated with neurological symptoms and persistent detection of ZIKV RNA in reproductive tissues^11^, we additionally assessed the brain and ovaries for the presence of infectious virus 7 dpi with ZIKV^BR^. However, we detected no virus in either of these tissues (Supplementary Fig. 1), suggesting that this virus is unable to widely disseminate in immunocompetent mice despite the reduced CD8 T cell response. Further analysis of the spleen and kidney for infectious virus 14 dpi with ZIKV^BR^ revealed that the virus was cleared from both of these tissues at this time point (Supplementary Fig. 1). Thus, ZIKV^BR^ induces a more sustained, but not chronic, infection in immunocompetent mice with replicative virus detected as late as day 7 post-infection in the spleen and kidney, which is cleared before day 14 post-infection.

### CD8 T cells are less activated and do not respond to the Env_294__–__302_ epitope following ZIKV^BR^ infection

**Fig. 2:**
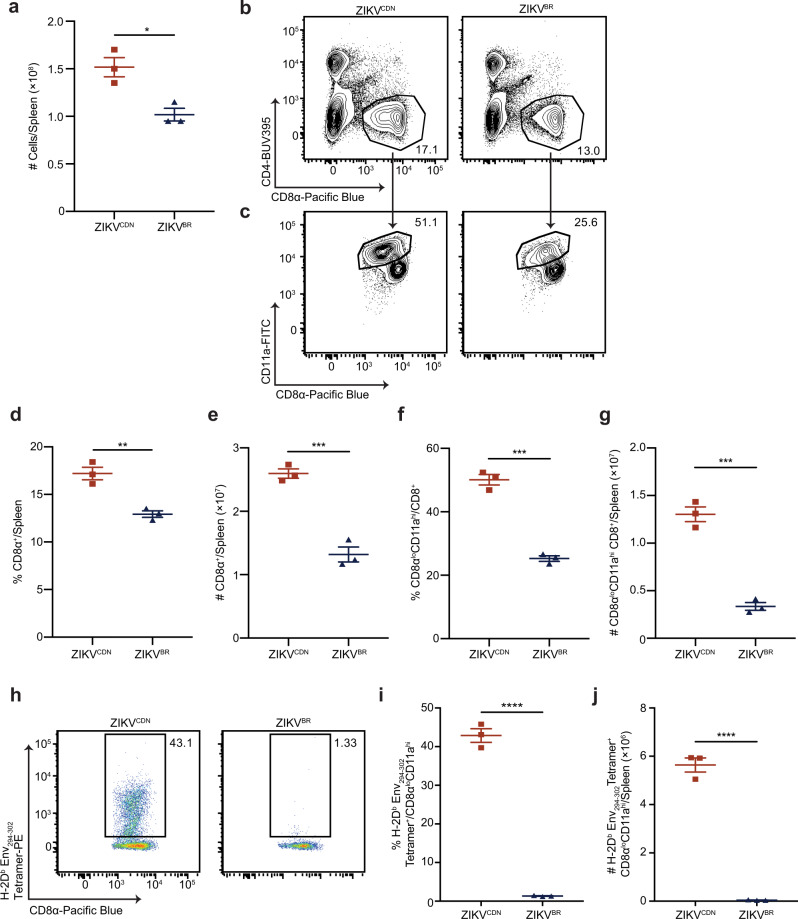
ZIKV^BR^ induces a less robust CD8 T cell response that does not respond to the immunodominant Env_294__–__302_ epitope. **a** Total spleen cellularity 7 dpi with ZIKV^CDN^ or ZIKV^BR^, *p* = 0.0146. **b**–**g** Representative flow cytometry plots (**b**), frequency (**d**), and number (**e**) of CD8α^+^ T cells 7 dpi with ZIKV^CDN^ or ZIKV^BR^. In **d** *p* = 0.0047 and in **e**, *p* = 0.0008. Representative flow cytometry plots (**c**), frequency (**f**), and number (**g**) of CD8α^lo^CD11a^hi^ CD8 T cells in the spleen 7 dpi with ZIKV^CDN^ or ZIKV^BR^. In **f** *p* = 0.0002 and in **g**, *p* = 0.0004. **h**–**j** Representative flow cytometry plots of H-2D^b^ Env_294__–__302_ tetramer-positive CD8α^lo^CD11a^hi^ CD8 T cells from ZIKV^CDN^ and ZIKV^BR^-infected mice (**h**), and frequency (**i**) and number (**j**) of H-2D^b^ Env_294__–__302_ tetramer-positive CD8α^lo^CD11a^hi^ CD8 T cells in the spleen 7 dpi with ZIKV^CDN^ or ZIKV^BR^. In **i** *p* = 0.000019 and in **j**, *p* = 0.000045. All data are representative of four independent experiments with *n* = 3 mice per group and are shown as mean ± SEM. All data were analyzed by a two-tailed, unpaired Student’s *t*-test. *p < 0.05, **p < 0.01, ***p < 0.001, and ****p < 0.0001. Red squares = ZIKV^CDN^-infected mice, blue triangles = ZIKV^BR^-infected mice. Source data are provided as a Source Data file.

Since our data demonstrated an overall defect in the CD8 T cell response to ZIKV^BR^, we next sought to further analyze this phenotype in the spleen 7 dpi. Our data demonstrate an overall decrease in total spleen cellularity following ZIKV^BR^ infection (Fig. 2a). In addition, ZIKV^BR^ induced significantly less total CD8 T cell expansion, as the frequency and number of CD8 T cells were significantly lower than what we observed following infection with ZIKV^CDN^ (Fig. 2b, d, e). Similar to our observations in the blood (Fig. 1a, b), both the frequency and number of CD8α^lo^CD11a^hi^ CD8 T cells were severely reduced during ZIKV^BR^ infection, compared to infection with ZIKV^CDN^ (Fig. 2c, f, g). Thus, the overall accumulation of cells in the spleen is reduced following ZIKV^BR^ infection, which corresponds to a significantly reduced induction of an antigen-experienced CD8 T cell response.

Previously, we and others identified Env_294–302_ as an immunodominant CD8 T cell epitope in the ZIKV envelope protein^22,24,25^. Therefore we tested the functionality of the CD8 T cell response to this epitope following each infection by analyzing the capacity of CD8α^lo^CD11a^hi^ CD8 T cells to produce the effector cytokine IFN-γ following stimulation with Env_294__–__302_ peptide. While we observed detectable IFN-γ production by CD8α^lo^CD11a^hi^ CD8 T cells following infection with ZIKV^CDN^, we observed no IFN-γ response to this peptide following ZIKV^BR^ infection (Supplementary Fig. 2). In addition, we did not detect Env_294__–__302_-specific CD8 T cells by H-2D^b^ class I MHC (MHC-I) tetramer (H-2D^b^ Env_294__–__302_) staining in ZIKV^BR^-infected mice, despite a robust Env_294–302_-specific response in ZIKV^CDN^-infected mice (Fig. 2h–j). In addition to the immunodominant Env_294–302_ epitope, several other immunogenic ZIKV peptides have been identified^24^. Thus, we tested a panel of 9 immunogenic peptides from ZIKV structural and NS proteins to determine their capacity to elicit IFN-γ production by CD8α^lo^CD11a^hi^ CD8 T cells. While we observed variable responsiveness to 7 of the epitopes, ZIKV^CDN^ infection elicited a consistent subdominant response above background following restimulation with PrM_169__–__177_ and NS5_2993__–__3000_ (Supplementary Fig. 3). Meanwhile, ZIKV^BR^ infection failed to induce a consistent response against any of the peptides tested (Supplementary Fig. 3). These data indicate that ZIKV^BR^ does not induce a CD8 T cell response against the immunodominant Env_294__–__302_ epitope and causes global changes in the capacity of CD8 T cells to recognize ZIKV-derived epitopes, despite the absence of any amino acid differences in these epitopes.

One possible explanation for the decreased CD8 T cell response to ZIKV^BR^ infection could include differences in antigen load following infection. Since we detected less ZIKV^BR^ RNA in the spleen 12 hpi compared to ZIKV^CDN^ (Fig. 1e), this difference could limit antigen availability for activating CD8 T cell responses. To address this question, we conducted a dose-dependence experiment, in which we infected mice with either 1 × 10^5^ PFU or 1 × 10^6^ PFU of ZIKV^CDN^, or 1 × 10^6^ PFU or 5 × 10^6^ PFU of ZIKV^BR^ (thereby reducing the potential antigen dose for ZIKV^CDN^ while increasing it for ZIKV^BR^). Between varying doses of the same virus, we observed no impact on either the total CD8α^lo^CD11a^hi^ CD8 T cell response nor the Env_294–302_-specific CD8 T cell response (Supplementary Fig. 4). Further, when comparing mice infected with 1 × 10^5^ PFU of ZIKV^CDN^ to those infected with 5 × 10^6^ PFU of ZIKV^BR^, with the latter group receiving an inoculation 50 times larger than the former, we still observed the same large reduction in both the total CD8α^lo^CD11a^hi^ CD8 T cell response and the Env_294–302_-specific CD8 T cell response (Supplementary Fig. 4). Thus, our data demonstrate that antigen load likely is not responsible for the reduced magnitude of the CD8 T cell response to ZIKV^BR^ infection, nor the lack of an Env_294__–__302_-specific CD8 T cell response.

**Fig. 3:**
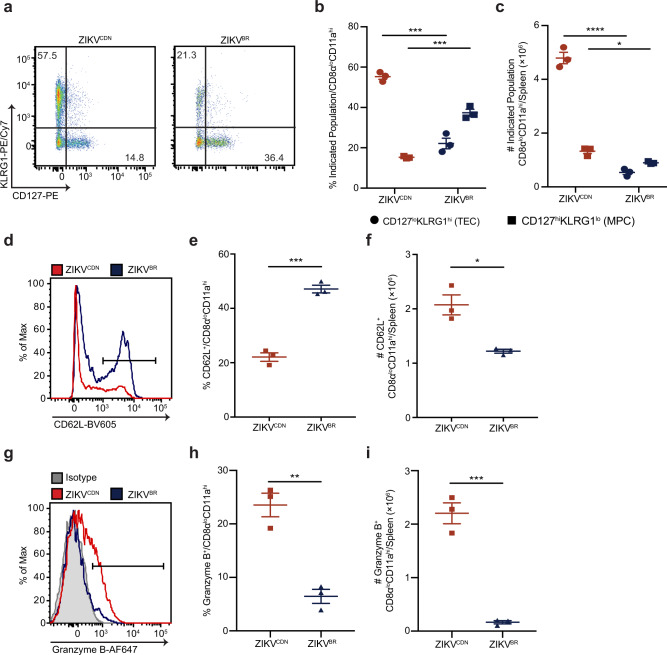
CD8 T cells present a less activated phenotype following ZIKV^BR^ infection. **a**–**c** Representative flow cytometry plots of CD127 and KLRG1 expression by CD8α^lo^CD11a^hi^ CD8 T cells from ZIKV^CDN^ and ZIKV^BR^-infected mice (**a**), and frequency (**b**) and number (**c**) of CD127^lo^KLRG1^hi^ CD8α^lo^CD11a^hi^ CD8 T cells (circles; TEC) and CD127^hi^KLRG1^lo^ CD8α^lo^CD11a^hi^ CD8 T cells (squares; MPC) in the spleen 7 dpi with ZIKV^CDN^ or ZIKV^BR^. In **b** TEC *p* = 0.0004 and MPC *p* = 0.0002. In **c** TEC *p* = 0.000049 and MPC *p* = 0.0103. **d**–**f** Representative histogram (**d**), frequency (**e**) and number (**f**) of CD62L^+^ CD8α^lo^CD11a^hi^ CD8 T cells in the spleen 7 dpi with ZIKV^CDN^ or ZIKV^BR^. Line on histogram indicates gating strategy used to identify CD62L^+^ cells. In **e** *p* = 0.0003 and in **f** *p* = 0.01. **g**–**i** Representative histogram (**g**), frequency (**h**), and number (**i**) of granzyme B^+^ CD8α^lo^CD11a^hi^ CD8 T cells in the spleen 7 dpi with ZIKV^CDN^ or ZIKV^BR^. Shaded histogram indicates isotype control. Line on histogram indicates gating strategy used to identify granzyme B^+^ cells. In **h** *p* = 0.0026 and in **i**, *p* = 0.0005. All data are representative of two independent experiments with *n* = 3 mice per group and are shown as mean ± SEM. All data were analyzed by a two-tailed, unpaired Student’s *t*-test. *p < 0.05, **p < 0.01, ***p < 0.001, and ****p < 0.0001. Red symbols = ZIKV^CDN^-infected mice, blue symbols = ZIKV^BR^-infected mice. Source data are provided as a Source Data file.

To assess CD8 T cell activation following ZIKV^BR^ infection, we analyzed the phenotype of total antigen-experienced CD8 T cells in the spleen at the peak of the T cell response 7 dpi. Following infection, effector CD8 T cells can be broadly classified as CD127^hi^KLRG1^lo^ memory precursor cells (MPCs), which preferentially survive contraction and seed the memory T cell pool, and CD127^lo^KLRG1^hi^ terminal effector cells (TECs), which are critical for the acute immune response, and largely die via apoptosis following peak expansion^33^. Following infection with ZIKV^CDN^, the majority of CD8α^lo^CD11a^hi^ CD8 T cells expressed a TEC phenotype, while more CD8α^lo^CD11a^hi^ CD8 T cells expressed a MPC phenotype following ZIKV^BR^ infection (Fig. 3a–c). We next examined the expression of CD62L (L-selectin), a lymph node homing receptor that is downregulated following TCR stimulation and is typically expressed by naive and central memory T cells, but not effector T cells^34^. A higher frequency and number of CD8α^lo^CD11a^hi^ CD8 T cells retained cell surface expression of CD62L during ZIKV^BR^ infection compared to ZIKV^CDN^ infection (Fig. 3d–f), suggesting a less activated phenotype. Finally, a significantly smaller frequency and number of CD8α^lo^CD11a^hi^ CD8 T cells responding to ZIKV^BR^ infection were positive for expression of the key cytolytic molecule granzyme B than those responding to ZIKV^CDN^ infection (Fig. 3g–i), suggesting they have reduced cytolytic capacity. Together, these data demonstrate that the small number of antigen-experienced CD8 T cells induced by ZIKV^BR^ infection present a less activated phenotype than those induced by ZIKV^CDN^ infection.

### Reduced dendritic cell activation and IFN-I production following ZIKV^BR^ infection do not impact CD8 T cell proliferation

**Fig. 4:**
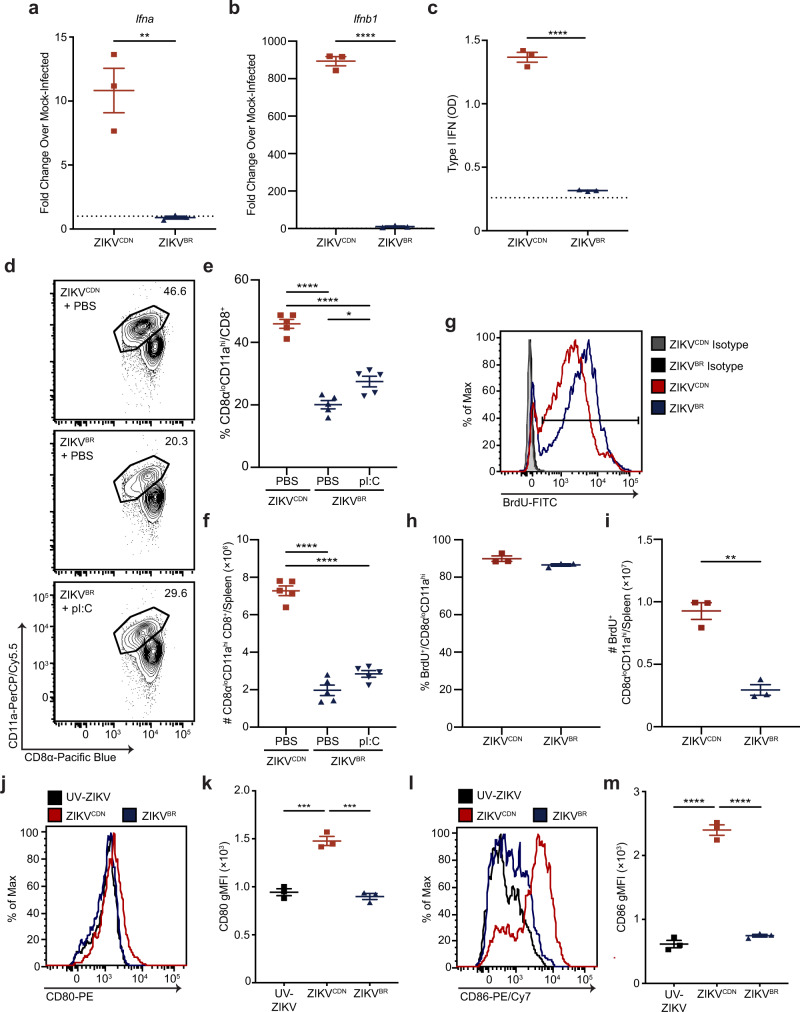
Reduced IFN-I production and dendritic cell activation following ZIKV^BR^ infection do not impact antigen-experienced CD8 T cell division. **a**, **b** *Ifna* (**a**) and *Ifnb1* (**b**) mRNA expression in the spleen were analyzed by RT-qPCR 12 hpi with ZIKV^CDN^ or ZIKV^BR^, or mock-infection. Data are expressed as fold change over expression in mock-infected mice 12 hpi. Dotted line indicates a fold change of 1. Data are representative of two independent experiments with *n* = 3 mice per group, and were analyzed by two-tailed, unpaired Student’s *t*-test. In **a** *p* = 0.0047 and in **b** *p* = 0.000004. **c** Total bioactive IFN-I were analyzed in the serum 12 hpi with ZIKV^CDN^ or ZIKV^BR^ using the B16-blue reporter cell line. Dotted line indicates average OD in the serum of mice 12 h post-mock infection. Data are representative of two independent experiments with *n* = 3 mice per group, and were analyzed by two-tailed, unpaired Student’s *t*-test, *p* = 0.000011. **d**–**f** Representative flow cytometry plots of CD8α^lo^CD11a^hi^ CD8 T cells from ZIKV^CDN^- or ZIKV^BR^-infected mice treated with PBS 2 and 3 dpi (**d**), and from ZIKV^BR^-infected mice treated with pI:C 2 and 3 dpi. Frequency (**e**) and number (**f**) of CD8α^lo^CD11a^hi^ CD8 T cells in the spleen 7 dpi with ZIKV^CDN^ or ZIKV^BR^, after treatment with either PBS or pI:C 2 and 3 dpi. Data are representative of two independent experiments with *n* = 5 mice per group and were analyzed by one-way ANOVA with Tukey’s post-test for multiple comparisons. In **e** ZIKV^CDN^ + PBS versus ZIKV^BR^ + PBS *p* = 0.0000001, ZIKV^CDN^ + PBS versus ZIKV^BR^ + pI:C *p* = 0.0000041, ZIKV^BR^ + PBS versus ZIKV^BR^ + pI:C *p* = 0.0112. In **f** ZIKV^CDN^ + PBS versus ZIKV^BR^ + PBS *p* = 0.000000008, ZIKV^CDN^ + PBS versus ZIKV^BR^ + pI:C *p* = 0.000000059. **g**–**i** Representative histogram (**g**), frequency (**h**), and number (**i**) of BrdU^+^ CD8α^lo^CD11a^hi^ CD8 T cells in the spleen 7 dpi with ZIKV^CDN^ or ZIKV^BR^. Line on histogram indicates gating strategy used to identify BrdU^+^ cells. Data are representative of two independent experiments with *n* = 3 mice per group, and were analyzed by two-tailed, unpaired Student’s *t*-test. In **i** *p* = 0.0013. **j**–**m** Representative histograms and geometric mean fluorescence intensity (gMFI) of CD80 (**j**, **k**) and CD86 (**l**, **m**) expression on CD3^−^CD19^−^NK1.1^−^ CD11c^+^MHC-II^+^ splenic DCs 2 dpi with ZIKV^CDN^ or ZIKV^BR^, or injection with an equivalent dose of UV-ZIKV. Data are representative of two independent experiments with *n* = 3 mice per group, and were analyzed by one-way ANOVA with Tukey’s post-test of multiple comparisons. In **k** ZIKV^CDN^ versus UV-ZIKV *p* = 0.0002 and ZIKV^CDN^ versus ZIKV^BR^ *p* = 0.0001. In **m** ZIKV^CDN^ versus UV-ZIKV *p* = 0.000002 and ZIKV^CDN^ versus ZIKV^BR^ *p* = 0.000002. All data are shown as mean ± SEM. *p < 0.05, **p < 0.01, ***p < 0.001, and ****p < 0.0001. Red squares and red lines = ZIKV^CDN^-infected mice, blue triangles and blue lines = ZIKV^BR^-infected mice, black squares and black lines = UV-ZIKV-injected mice, gray lines = isotype controls. Source data are provided as a Source Data file.

Inflammatory cytokines such as IFN-I provide a crucial signal during CD8 T cell priming that is important for their activation, accumulation, acquisition of effector functions, and capacity to respond to low levels of antigen^35–37^. In contrast, cytokines such as IL-10 have broad immunoregulatory effects that dampen inflammatory responses, including reducing CD8 T cell accumulation and function^38–40^. Further, Asian lineage ZIKV infection of human peripheral blood mononuclear cells has been shown to induce more IL-10 production than infection with African lineage isolates^41^. We, therefore, hypothesized that the blunted CD8 T cell response to ZIKV^BR^ could be the result of either increased production of anti-inflammatory cytokines such as IL-10, or reduced production of inflammatory cytokines such as IFN-I. To determine whether IL-10 production during ZIKV^BR^ infection contributed to the blunted CD8 T cell response, we infected WT and *Il10*^*−/−*^ mice with ZIKV^BR^ and assessed the impact of IL-10 deficiency on the antigen-experienced CD8 T cell response 7 dpi. We observed no differences in the frequency or number of CD8α^lo^CD11a^hi^ CD8 T cells between WT and *Il10*^*−/−*^ mice, indicating that IL-10 does not play a role in blunting the CD8 T cell response to ZIKV^BR^ infection (Supplementary Fig. 5). We next assessed *Ifna* (IFN-α) and *Ifnb1* (IFN-β) mRNA expression in the spleen 12, 24, and 72 hpi with each ZIKV isolate by RT-qPCR. In contrast to robust expression following ZIKV^CDN^ infection, ZIKV^BR^ infection did not induce IFN-α/β mRNA expression above mock-infection levels at 12 hpi (Fig. 4a, b). IFN-α/β mRNA expression remained elevated in ZIKV^CDN^-infected mice at 24 hpi and returned to baseline 72 hpi, with minimal expression detected at either time point in ZIKV^BR^-infected mice (Supplementary Fig. 6). Furthermore, analysis of bioactive IFN-I protein levels 12, 24, and 72 hpi, as well as 7 dpi, with each ZIKV isolate revealed the presence of IFN-I in the serum was drastically reduced following infection with ZIKV^BR^, and remained low at all time points analyzed (Fig. 4c and Supplementary Fig. 6). Following ZIKV^CDN^ infection, IFN-I protein levels mirrored the RT-qPCR results, with robust production 12 and 24 hpi, which returned to baseline 72 hpi and 7 dpi (Fig. 4c and Supplementary Fig. 6). Analysis of the IFN-stimulated genes (ISGs) *Ifitm3*, *Irf7*, *Isg15,* and *Oas1a* at 12, 24, and 72 hpi demonstrated a consistent trend for increased expression following ZIKV^CDN^ infection (Supplementary Fig. 6). Although not to the same extent as ZIKV^CDN^, ZIKV^BR^ did induce ISG expression above mock infection, suggesting some IFN-I signaling occurs following infection, which is consistent with the detection of low levels of bioactive IFN-I following infection at 12 hpi (Fig. 4c and Supplementary Fig. 6). However, even at 7 dpi when viral load is high in the spleen and kidney (Fig. 1e–g), we observed little to no IFN-I production in ZIKV^BR^-infected mice. These data demonstrate that IFN-I production is poorly induced by ZIKV^BR^ infection and that this is not simply a delay in the induction of the response, but rather a sustained defect despite continued viral replication. Thus, our data suggest that while IL-10 does not contribute to the reduced antigen-experienced CD8 T cell response to ZIKV^BR^ infection, this defect may be due to a reduced IFN-I response.

Since ZIKV^BR^ poorly induces IFN-I and ISG production, we reasoned that providing a source of IFN-I could restore the magnitude antigen-experienced CD8 T cell response. To test this, we infected mice with ZIKV^CDN^ or ZIKV^BR^ and determined the impact of treating ZIKV^BR^-infected mice on days 2 and 3 post-infection with the toll-like receptor (TLR)−3 agonist polyinosinic:polycytidylic acid (pI:C) or murine rIFN-β on the CD8α^lo^CD11a^hi^ CD8 T cell response 7 dpi. Although we observed a small, but significant increase in the frequency of CD8α^lo^CD11a^hi^ CD8 T cells in pI:C-treated mice compared to control-treated animals, neither treatment had an impact on the total number of CD8α^lo^CD11a^hi^ CD8 T cells in ZIKV^BR^-infected mice (Fig. 4d–f and Supplementary Fig. 7). Thus, reduced IFN-I production following ZIKV^BR^ infection is not sufficient to explain the abrogated antigen-experienced CD8 T cell response.

The lower accumulation of antigen-experienced CD8 T cells following ZIKV^BR^ infection could be due to a reduction in the proliferative capacity of CD8 T cells. To address this question, mice were infected with either isolate, pulsed with BrdU, and maintained on BrdU drinking water for the duration of the experiment. As cells divide, BrdU becomes incorporated into the DNA in the place of thymidine, and the amount of BrdU incorporation may be used as a read-out of proliferation. We observed an equivalent frequency of BrdU^+^ CD8α^lo^CD11a^hi^ CD8 T cells following either infection, indicating that proliferation is not impacted by ZIKV^BR^ infection (Fig. 4g–i). In addition, intracellular staining for Ki67, a marker of cell cycle entry, in CD8α^lo^CD11a^hi^ CD8 T cells was also equivalent between ZIKV^CDN^ and ZIKV^BR^ infection (Supplementary Fig. 8). These data indicate that the decreased magnitude of the CD8α^lo^CD11a^hi^ CD8 T cell response to ZIKV^BR^ is not due to a defect in proliferation and suggest that once CD8 T cells are primed, they are capable of proliferating normally.

Since our data suggest that the reduced magnitude of the CD8 T cell response to ZIKV^BR^ is not due to a defect in proliferation, we examined whether there was a defect in T cell priming. Thus, we injected mice with UV-ZIKV or infected with ZIKV^CDN^ or ZIKV^BR^ and analyzed DC activation 2 dpi based on cell surface expression of the co-stimulatory molecules CD80 and CD86. Although both markers were significantly upregulated on DCs following ZIKV^CDN^ infection, we did not observe significant upregulation following ZIKV^BR^ infection, which suggests DCs are poorly activated by ZIKV^BR^ infection (Fig. 4j–m). These data suggest that although the level of DC activation observed following ZIKV^BR^ infection is sufficient to induce comparable CD4 T cell responses (Fig. 1c, d), it is not sufficient to fully activate CD8 T cells. However, reduced DC activation during ZIKV^BR^ infection is unlikely to explain the complete lack of a response against the Env_294__–__302_ epitope, suggesting that several mechanisms may be involved in the capacity of ZIKV^BR^ to counter immune responses.

### ZIKV^BR^ modulates cross-presentation and immunoproteasome activation to subvert antigen-specific CD8 T cell response

**Fig. 5:**
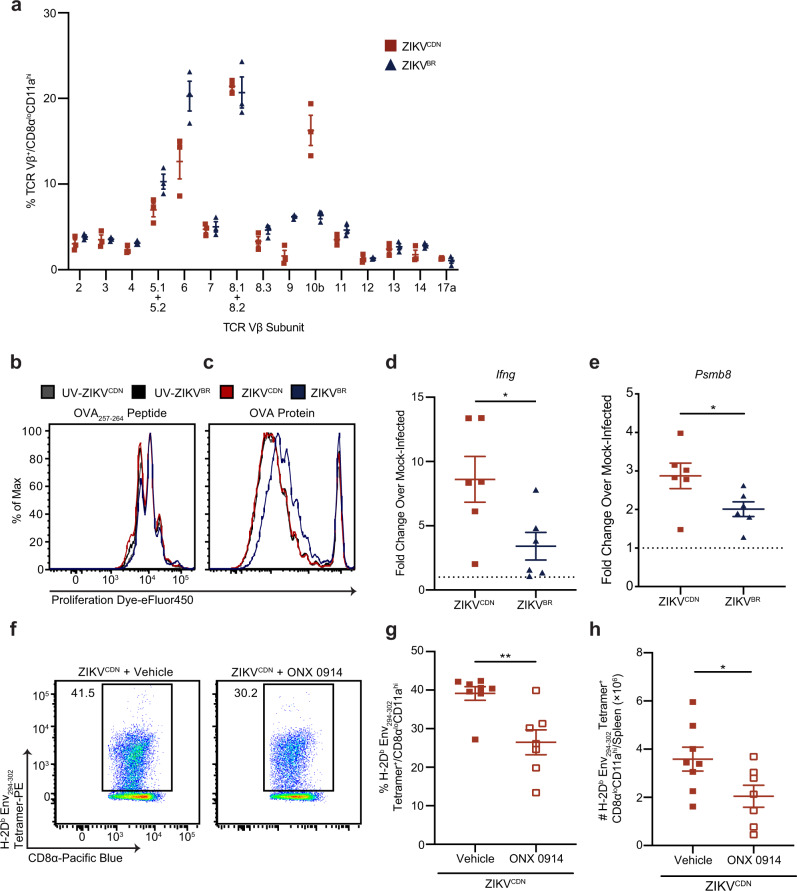
ZIKV^BR^ infection suppresses antigen cross-presentation and immunoproteasome induction. **a** TCR Vβ subunit usage among CD8α^lo^CD11a^hi^ CD8 T cells 7 dpi with ZIKV^CDN^ or ZIKV^BR^. Data are representative of two independent experiments with n = 3 mice per group. **b**, **c** Proliferation dye dilution in OT-I CD8 T cells 3 days following co-culture with BMDCs. Prior to co-culture with labeled OT-I CD8 T cells, DCs were infected with ZIKV^CDN^ or ZIKV^BR^, or UV-inactivated ZIKV^CDN^ or ZIKV^BR^, at a MOI of 5 for 6 h, followed by a 4-h incubation with LPS and either OVA_257__–__264_ peptide (**b**) or OVA protein (**c**). Data are representative of three independent experiments. **d** mRNA expression of *Ifng* in the spleen was analyzed by RT-qPCR 12 hpi with ZIKV^CDN^ or ZIKV^BR^, or mock-infection. Data are expressed as fold change over expression in mock-infected mice 12 hpi. Dotted line indicates a fold change of 1. Data are pooled from two independent experiments with *n* = 3 mice per group and were analyzed by two-tailed, unpaired Student’s *t*-test, *p* = 0.0313. **e** mRNA expression of *Psmb8* in the spleen was analyzed by RT-qPCR 12 hpi with ZIKV^CDN^ or ZIKV^BR^, or mock-infection. Data are expressed as fold change over expression in mock-infected mice 12 hpi. Dotted line indicates a fold change of 1. Data are pooled from two independent experiments with *n* = 3 mice per group and were analyzed by two-tailed, unpaired Student’s *t*-test, *p* = 0.0466. **f**–**h** Representative flow cytometry plots of H-2D^b^ Env_294__–__302_ tetramer-positive CD8α^lo^CD11a^hi^ CD8 T cells from ZIKV^CDN^-infected mice following treatment with vehicle control or ONX 0914 (**f**). Frequency (**g**) and number (**h**) of H-2D^b^ Env_294__–__302_ tetramer-positive CD8α^lo^CD11a^hi^ CD8 T cells in the spleen 7 dpi with ZIKV^CDN^. Mice were treated s.c. with vehicle control or ONX 0914 prior to infection. Data are pooled from two independent experiments with *n* = 3 (first experiment), *n* = 4 (ONX 0914 treated, second experiment), or *n* = 5 mice per group (vehicle-treated, second experiment), and were analyzed by two-tailed, unpaired Student’s *t*-test. In **g** *p* = 0.0034 and in **h** *p* = 0.0414. All data are shown as mean ± SEM. *p < 0.05, and **p < 0.01. Red squares and red lines = ZIKV^CDN^-infected mice or BMDC, blue triangles and blue lines = ZIKV^BR^-infected mice or BMDC, black lines = UV- ZIKV^BR^ inoculated BMDCs, gray lines = UV-ZIKV^CDN^ inoculated BMDCs. Source data are provided as a Source Data file.

Antigen-experienced CD8 T cells do not respond to the immunodominant Env_294__–__302_ epitope following ZIKV^BR^ infection (Fig. 2h–j and Supplementary Fig. 2). As such, we next investigated whether this was reflective of broader changes to the CD8 T cell pool that is activated following ZIKV^BR^ infection. To address this question, we analyzed TCR Vβ subunit usage among antigen-experienced CD8 T cells following each infection. We observed that the frequencies of most Vβ subunits are comparable regardless of the infection (Fig. 5a). However, a higher frequency of CD8α^lo^CD11a^hi^ CD8 T cells use TCR Vβ10b following ZIKV^CDN^ infection, whereas a higher frequency of Vβ6 and Vβ9-expressing CD8α^lo^CD11a^hi^ CD8 T cells were observed following ZIKV^BR^ infection (Fig. 5a). Together, these data suggest that ZIKV^BR^ infection is associated with broad changes to the CD8 T cell repertoire, which could be due to alterations in priming of the CD8 T cell response.

Initial priming of a naive CD8 T cell requires the presentation of cognate antigen in the context of MHC-I, typically by mature DCs. The presentation of exogenously acquired antigen by a DC occurs via a mechanism known as cross-presentation^42^. To determine whether ZIKV^BR^ is capable of altering cross-presentation to subvert priming of the CD8 T cell response, we analyzed the capacity of each ZIKV isolate to disrupt cross-presentation of ovalbumin (OVA) by bone marrow-derived DCs (BMDCs). Briefly, BMDCs were infected with either ZIKV^CDN^ or ZIKV^BR^ for 6 h (incubation with an equivalent amount of UV-inactivated ZIKV^CDN^ or ZIKV^BR^ was used as controls). Following infection, BMDCs were incubated with LPS, so that their capacity to stimulate CD8 T cell responses would be independent of each virus’ capacity to activate the BMDCs (Supplementary Fig. 9), as well as OVA_257__–__264_ peptide or OVA protein. Four hours later, proliferation dye-labeled TCR-transgenic OT-I CD8 T cells (specific for the OVA_257–264_ epitope) were added to BMDCs from each infection group and OT-I proliferation was assessed 3 days later. We observed no differences in OT-I proliferation following incubation with BMDCs that were pulsed with OVA_257__–__264_ peptide, regardless of the virus isolate or UV-inactivated virus (Fig. 5b). However, when BMDCs were pulsed with OVA protein, we observed reduced OT-I proliferation when the BMDCs had been infected with ZIKV^BR^, and not ZIKV^CDN^, or either UV-inactivated virus (Fig. 5c). Importantly, these differences were not due to a change in the capacity of BMDCs to uptake OVA protein (Supplementary Fig. 9). Thus, these data suggest that ZIKV^BR^ is able to disrupt cross-presentation, independently of impacting antigen uptake, to evade CD8 T cell responses.

To further elucidate the mechanism by which ZIKV^BR^ subverts CD8 T cell immunity, we asked whether ZIKV^BR^ infection also led to alterations in the generation of immunogenic epitopes. The immunoproteasome is an alternative form of the constitutive proteasome that is induced during immune responses to generate antigens for presentation on MHC-I^43^. Exposure to inflammatory cytokines such as IFN-γ leads to the production of large molecular protein (LMP)2, LMP10 and LMP7, which substitute for the β1, β2, and β5 subunits of the constitutive proteasome, respectively^43^. Given the role of IFN-γ in immunoproteasome induction, we first assessed *Ifng* (IFN-γ) mRNA expression in the spleen 12 hpi with ZIKV^CDN^ or ZIKV^BR^ by RT-qPCR. Although ZIKV^BR^ induced detectable IFN-γ transcripts, we observed significantly higher induction of IFN-γ following infection with ZIKV^CDN^ (Fig. 5d). In line with this observation, ZIKV^BR^ infection induced significantly less mRNA expression of *Psmb8* (LMP7) in the spleen 12 hpi compared to ZIKV^CDN^ infection (Fig. 5e), suggesting activation of the immunoproteasome is impaired during ZIKV^BR^ infection. To determine whether the immunoproteasome plays a functional role in the generation of the Env_294–302_-specific CD8 T cell response during ZIKV^CDN^ infection, we treated ZIKV^CDN^-infected mice with either vehicle control or ONX 0914, an LMP7-selective inhibitor that has previously been used to ameliorate disease progression in mouse models of autoimmune disease^44–47^. We observed that ONX 0914 treatment caused a significant reduction in both the frequency and number of Env_294–302_-specific CD8 T cells following ZIKV^CDN^ infection (Fig. 5f–h). Thus, generation of the Env_294–302_-specific CD8 T cell response is at least partially dependent on the immunoproteasome. This suggests that failure to induce the immunoproteasome during ZIKV^BR^ infection could be responsible for the lack of a response against the Env_294–302_ epitope.

### ZIKV^BR^ actively disrupts the antigen-specific CD8 T cell response

**Fig. 6:**
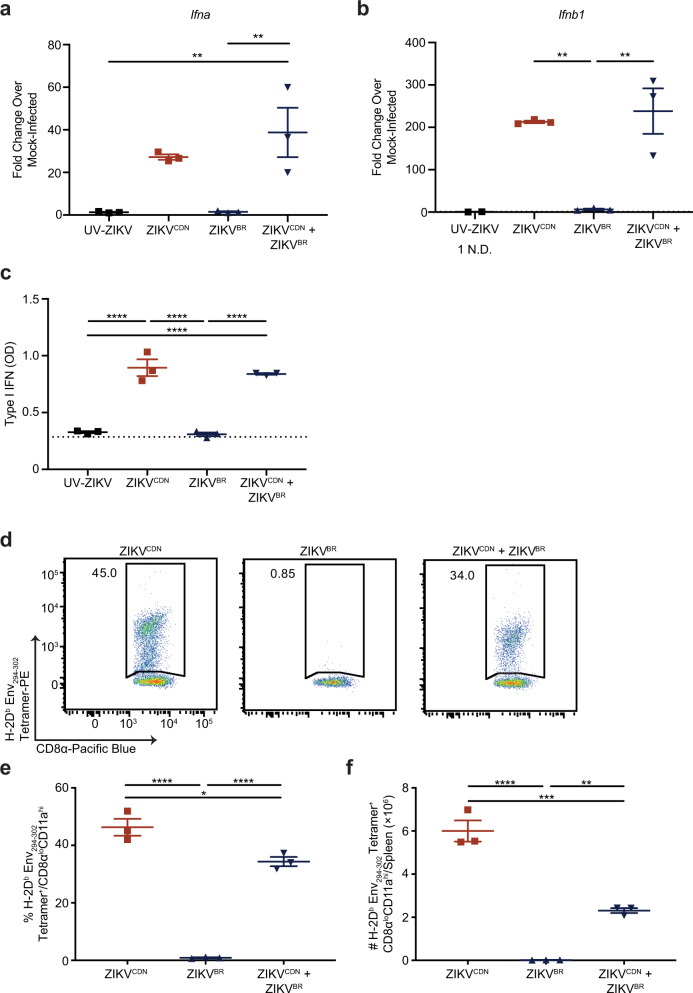
ZIKV^BR^ infection actively suppresses the Env_294__–__302_-specific CD8 T cell response during co-infection. **a**, **b** *Ifna* (**a**) and *Ifnb1* (**b**) mRNA expression in the spleen were analyzed by RT-qPCR 12 hpi with ZIKV^CDN^ or ZIKV^BR^, co-infection, injection with UV-ZIKV, or mock-infection. Data are expressed as fold change over expression in mock-infected mice 12 hpi. N.D. indicates no expression was detected by RT-qPCR. In **a** UV-ZIKV versus ZIKV^CDN^ + ZIKV^BR^ *p* = 0.0082 and ZIKV^BR^ versus ZIKV^CDN^ + ZIKV^BR^ *p* = 0.0085. In **b** ZIKV^CDN^ versus ZIKV^BR^ *p* = 0.008 and ZIKV^BR^ versus ZIKV^CDN^ + ZIKV^BR^ *p* = 0.0045. **c** Total bioactive IFN-I were analyzed in the serum 12 hpi with ZIKV^CDN^ or ZIKV^BR^, co-infection, injection with UV-ZIKV, or mock-infection using the B16-blue reporter cell line. Dotted line indicates average OD in the serum of mice 12 h post-mock infection. UV-ZIKV versus ZIKV^CDN^ *p* = 0.000029, UV-ZIKV versus ZIKV^CDN^ + ZIKV^BR^ *p* = 0.00006, ZIKV^BR^ versus ZIKV^CDN^ *p* = 0.000022, ZIKV^BR^ versus ZIKV^CDN^ + ZIKV^BR^ *p* = 0.000046. **d**–**f** Representative flow cytometry plots of H-2D^b^ Env_294__–__302_ tetramer-positive CD8α^lo^CD11a^hi^ CD8 T cells from ZIKV^CDN^-, ZIKV^BR^- or co-infected mice (**d**). Frequency (**e**) and number (f) of H-2D^b^ Env_294__–__302_ tetramer-positive CD8α^lo^CD11a^hi^ CD8 T cells in the spleen 7 dpi with ZIKV^CDN^ or ZIKV^BR^, or co-infection. In **e** ZIKV^CDN^ versus ZIKV^BR^ *p* = 0.000007, ZIKV^CDN^ versus ZIKV^CDN^ + ZIKV^BR^ *p* = 0.0112, ZIKV^BR^ versus ZIKV^CDN^ + ZIKV^BR^ *p* = 0.000044. In **f** ZIKV^CDN^ versus ZIKV^BR^ *p* = 0.000016, ZIKV^CDN^ versus ZIKV^CDN^ + ZIKV^BR^ *p* = 0.0003, ZIKV^BR^ versus ZIKV^CDN^ + ZIKV^BR^ *p* = 0.0034. All data are representative of two independent experiments with *n* = 3 mice per group and are shown as mean ± SEM. All data were analyzed by one-way ANOVA with Tukey’s post-test of multiple comparisons. UV-ZIKV group in **b** was excluded from statistical analysis. *p < 0.05, **p < 0.01, ***p < 0.001, and ****p < 0.0001. Red squares = ZIKV^CDN^-infected mice, blue triangles = ZIKV^BR^-infected mice, inverted blue triangles = ZIKV^CDN^ + ZIKV^BR^ co-infected mice, black squares = UV-ZIKV-injected mice. Source data are provided as a Source Data file.

In addition to determining mechanisms of immune evasion by ZIKV^BR^, we also asked whether ZIKV^BR^ is actively suppressing immune responses or passively avoiding detection. To address this question, we reasoned that if ZIKV^BR^ is actively disrupting a pathway, and doing so in a dominant manner, it would be capable of disrupting the response induced by ZIKV^CDN^ in the context of co-infection. Thus, we infected mice with ZIKV^CDN^ or ZIKV^BR^ alone, or co-infected mice with both isolates, and assessed the impact of co-infection on IFN-I production (12 hpi) and the Env_294__–__302_-specific CD8 T cell response (7 dpi). Given that antigen-experienced CD8 T cells do not respond to this epitope during ZIKV^BR^ infection, this approach enabled us to analyze the impact of infection with ZIKV^BR^ on the ZIKV^CDN^-specific CD8 T cell response. We observed that co-infection did not reduce IFN-I production, at either the mRNA or protein level, compared to ZIKV^CDN^ infection alone, indicating that ZIKV^BR^ does not block IFN-I production in a dominant manner (Fig. 6a–c). However, we observed a significant reduction in both the frequency and number of Env_294__–__302_-specific CD8 T cells following co-infection, suggesting that ZIKV^BR^ actively disrupts the antigen-specific CD8 T cell response to ZIKV^CDN^ (Fig. 6d–f). Thus, these data demonstrate that ZIKV^BR^ modulates the CD8 T cell response through both active and passive mechanisms.

## Discussion

Since its initial isolation, ZIKV has emerged as a major global health concern, which retains the capacity to cause severe neurological symptoms in both adults and infants as it encounters naive host populations. As such, developing robust means of studying the immune response to infection, and how it may have changed in recent outbreaks, is key to developing novel vaccination and treatment strategies. Here, we have used our previously established model of ZIKV^CDN^ infection^22^ as a baseline to which we compared the immune response induced by one epidemic isolate, ZIKV^BR^. Although mice are infected i.v. in this model, which is less representative of the natural route of infection, this provides a detectable and consistent means of interrogating the immune response in a fully immunocompetent setting. Our data suggest that ZIKV^BR^ severely compromises several innate and adaptive protection mechanisms to establish a sustained, but not chronic, infection in immunocompetent mice. Further, infection with ZIKV^BR^ yields a severely blunted CD8 T cell response, with fewer antigen-experienced CD8 T cells, the absence of a CD8 T cell response to a key immunodominant epitope, and overall reduced CD8 T cell activation. Additionally, we have established that reduced cross-presentation and immunoproteasome induction following ZIKV^BR^ infection subvert generation of the response against the immunodominant Env_294__–__302_ epitope and other subdominant epitopes. Together, these data suggest the enhanced capacity of this particular ZIKV isolate to counter host immune defenses, which may provide insight into how this virus gained epidemic capacity associated with enhanced pathogenicity, although the broader implication of these findings remains to be confirmed with other epidemic isolates.

Although ZIKV^BR^ exhibits “slower” replication 12 hpi, by 5 and 7 dpi viral load increased in ZIKV^BR^-infected mice, while it is decreasing in ZIKV^CDN^-infected mice. Similar observations of slower initial virus replication leading to weaker CD8 T cell responses and longer detection of viral loads have been observed in a comparison of infection with several lymphocytic choriomeningitis virus (LCMV) strains in mice^48^. In humans, patients with slower-replicating hepatitis C virus isolates are associated with an increased likelihood of chronic persistence^48^. Importantly, our data strongly suggest that reduced antigen load is not the mechanism behind the reduced CD8 T cell response to ZIKV^BR^ infection, despite this initial lower level of virus RNA detection, as varying viral inoculum did not affect the magnitude of the T cell response. This viral immune evasion strategy is in contrast to what is typically observed during infection with LCMV clone 13 in mice, where enhanced virus replication is associated with T cell exhaustion and chronic infection^49–51^. Indeed, ZIKV^BR^ does not establish a chronic infection in WT mice and is cleared from the spleen and kidney by 14 dpi. Determining which immune mechanisms, in the absence of a robust CD8 T cell response, are responsible for this delayed viral clearance represents an intriguing question and will be the focus of future studies.

Persistent detection of ZIKV RNA has been reported in a number of human studies and case reports from recent ZIKV outbreaks^11,12,14,52–55^. A prospective cohort study from Brazil reported that the median time to clearance of viral RNA was 13 days POS in vaginal secretions, 15 days in serum, 11 days in urine, and 42 days in semen, although in 11% of men ZIKV RNA remained detectable in semen 90 days POS^12,14^. Since longitudinal studies from previous outbreaks were not conducted, it is not possible to definitively state whether this represents a newly acquired characteristic of ZIKV. However, for some patients, the presence of ZIKV RNA in bodily fluids such as the cerebrospinal fluid, aqueous humor, or semen correlate with previously unreported symptoms such as meningitis or meningoencephalitis, uveitis, and hematospermia, respectively^11,13,56–58^. Although we report sustained detection of ZIKV^BR^ RNA in the spleen, and infectious virus in the spleen and kidney 7 dpi, ZIKV^BR^ was not detected in neurological or reproductive tissues. Thus, while our findings support an important and valuable model to interrogate how ZIKV^BR^ counters innate and adaptive immune responses, and to study ZIKV immunity more broadly, it is less appropriate for studies of viral neuropathogenesis or sexual transmission.

Our data establish that, compared to ZIKV^CDN^, ZIKV^BR^ induces a severely reduced CD8 T cell response and that the few responding CD8 T cells present are less activated. In contrast to robust DC activation and IFN-I production observed following ZIKV^CDN^ infection, ZIKV^BR^ poorly activated DCs and induced little to no IFN-I at multiple time points post-infection. In spite of this deficit, exogenous induction or addition of IFN-I was not sufficient to restore CD8 T cell numbers. During ZIKV infection, T cell responses are largely considered to play an important and protective role^59^. Our group and others have demonstrated that ZIKV infection in immunocompetent mice can lead to induction of CD8 T cell immunity, featuring the production of key effector cytokines such as IFN-γ or TNF-α, and the cytolytic molecule granzyme B, suggesting they are important for ZIKV immunity^22,25^. Further, T cells were shown to protect immunocompetent mice from high viral loads in the central nervous system and severe disease following intracranial infection, as this protection was lost in T cell-deficient mice^60^. The correlation between reduced CD8 T cell activation and accumulation following ZIKV^BR^ infection and more sustained virus detection suggests CD8 T cells may play a protective role in this context. However, it has also been suggested that T cells are dispensable for ZIKV infection, as depletion of both CD4 and CD8 T cells prior to infection with a Brazilian ZIKV isolate led to only a small but significant weight loss compared to control-treated mice^61^. It is worth noting, however, that the IFN response was not analyzed in this study, so it remains possible that this isolate did not induce a robust IFN-I or CD8 T cell response, similar to our observations herein^61^.

Immunoproteasome activation and cross-presentation of antigens are important steps in the generation of a protective antigen-specific antiviral CD8 T cell response^42,43^. The importance of the immunoproteasome in generating CD8 T cell responses varies depending on the infection. During murine cytomegalovirus infection, nearly all CD8 T cell epitopes are dependent on LMP7 expression^62^. Conversely, the generation of CD8 T cell responses against LCMV infection is only partially dependent on the immunoproteasome, and requires deletion of all three subunits of the immunoproteasome in order to observe a major impact^62^. Our data establish the importance of immunoproteasome induction in ZIKV immunity, since following ZIKV^CDN^ infection, generation of the Env_294__–__302_-specific CD8 T cell response is at least partially dependent on LMP7. Further, ZIKV^CDN^ also induced subdominant PrM_169__–__177_ and NS5_2993__–__3000_-specific responses, which were not observed following ZIKV^BR^ infection. These were the only other two previously described epitopes that elicited a response in our model, which is likely due to differences between WT mice and the LysMCre^+^IFNAR^fl/fl^ mouse model of ZIKV infection^24^. Together, the absence of an Env_294__–__302_-specific response and the altered TCR Vβ repertoire during ZIKV^BR^ infection, along with our BMDC co-culture experiments support the idea that generation and presentation of immunogenic antigens is altered during infection with this isolate. Moreover, our co-infection data suggests that ZIKV^BR^ actively mediates this inhibition. This mechanism has not previously been associated with ZIKV infection, and it opens exciting questions as to which viral proteins mediate this inhibition, which mutations have conferred this capacity to ZIKV^BR^, and whether these mechanisms are broadly shared amongst epidemic isolates, which will be the focus of future investigations.

In conclusion, our findings demonstrate that, in contrast to ZIKV^CDN^, ZIKV^BR^ actively counters CD8 T cell immunity, which correlates with more sustained, but not chronic, infection in both the spleen and kidney of immunocompetent mice. Although we observed a comparable CD4 T cell response in the blood between the two infections, insufficient DC activation and reduced IFN-I production following ZIKV^BR^ infection are associated with a poorly activated CD8 T cell response. The lack of a response against the immunodominant Env_294__–__302_ epitope, the generation of which requires immunoproteasome activity, resulted in a severe reduction in the number of CD8 T cells responding to ZIKV^BR^ infection and an obvious change in the viral epitopes being targeted by the remaining CD8 T cells. It is important to note that our studies have only identified these mechanisms using one contemporary isolate of ZIKV and it remains possible that this is only representative of this isolate and not broadly conserved in all epidemic strains. This remains a possibility as phylogenetic studies have identified a distinct ZIKV lineage in the Bahia province of Brazil, where the isolate used in this study was isolated, although this particular isolate was not analyzed as part of that study^63^. Thus, in future studies, it will be important to determine whether all or some of these immune evasion mechanisms are conserved among additional epidemic ZIKV isolates, including currently circulating isolates. Such studies could identify whether the mechanisms described herein are broadly conserved, subject to regional specificities, or even whether they independently emerged in distinct locations. This could also help determine which of the 13 amino acid mutations mediate these viral immune evasion mechanisms. Nonetheless, our data suggest that even within the Asian lineage, at least one ZIKV isolate has acquired an enhanced capacity to subvert host immunity, with a particularly severe impact on the CD8 T cell response. Reduced CD8 T cell responses may be related to longer-lasting infections with ZIKV, which could contribute to sustained detection of viral RNA and pathogenesis observed during recent outbreaks.

## Methods

### Cell Lines

Vero cells (African Green monkey kidney epithelial cells, provided by Steven Varga, University of Iowa) were cultured in DMEM (Wisent) supplemented with 10% heat-inactivated FBS (Wisent), 1% l-glutamine (Wisent), 1% Penicillin–Streptomycin (Wisent) and 1% non-essential amino acids (Sigma) at 37 °C and 5% CO_2_. B16-Blue cells (provided by Maziar Divangahi, McGill University) were cultured in RPMI-1640 supplemented with 10% heat-inactivated FBS, 0.5% Penicillin–Streptomycin, 1% l-glutamine, and 100 μg/mL Normocin (InvivoGen). Cells were passaged three times before the addition of selection antibiotic (Zeocin, InvivoGen) at a final concentration of 100 μg/mL.

### Viruses

Low passage (p.4) ZIKV^CDN^ (PLCal_ZV, Genbank accession KF993678) derived from a ZIKV-infected traveler returning to Canada was provided by Gary Kobinger and David Safronetz (National Microbiology Laboratory and Public Health Agency of Canada)^26^. Low passage (p.4) ZIKV^BR^ (HS-2015-BA-01, Genbank accession KX520666) isolated from a symptomatic patient in August 2015 in Salvador, Bahia was provided by Mauro Teixeira (Universidade Federal de Minas Gerais). The stock was originally passaged three times in C6/36 mosquito cells and once in Vero cells. To propagate ZIKV stocks, 6 × 10^6^ Vero cells were seeded in 150 mm × 25 mm dishes (Sigma), and 24 h later infected at a multiplicity of infection (MOI) of 1 for 2 h in unsupplemented EMEM. After 2 h, infection media was removed and replaced with 15 mL of DMEM supplemented with 2% heat-inactivated FBS, 1% l-glutamine, 1% non-essential amino acids, 1% Penicillin–Streptomycin, and 15 mM Hepes. Supernatants from both mock-infected as well as ZIKV-infected cells were harvested after 48–72 hpi, centrifuged for 10 min at 3000 × *g*, and aliquoted. Viral stocks were titered by plaque assay on Vero cells. Briefly, viral stocks were serially diluted (10-fold) in EMEM (Wisent) and 100 μL of each dilution was used to infect confluent monolayers of Vero cells in 1 mL of unsupplemented EMEM. At 2 hpi, infection media was removed, and cells were overlaid with 1.2% carboxymethyl cellulose (Sigma-Aldrich) and 2% heat-inactivated FBS in EMEM for 4 days prior to fixation for 1 h with an equal volume of 10% formaldehyde. Monolayers were rinsed gently with distilled water and stained for 30 min with 0.1% crystal violet prior to counting plaques. ZIKV^CDN^ or ZIKV^BR^ was UV-inactivated by transferring 1 mL of ZIKV into one well of a 6-well plate and exposing it to 3 Joules/cm^2^ of UV irradiation in a UVC 500 Crosslinker (Hoefer) as previously described^22^. UV-inactivation was verified by plaque assay.

### Mouse experiments

C57BL/6 mice (WT) were purchased from Charles River laboratories or bred at McGill University. *Il10*^*−/−*^ mice were originally purchased from The Jackson Laboratory and were subsequently bred in-house at McGill University. Mice with OT-I TCR-transgenic CD8 T cells (specific for OVA_257–264_ peptide) were previously described^64^. Infected mice were housed in biocontainment level 2 and all animal procedures were carried out in accordance with Canadian Council on Animal Care ethical guidelines and received ethical approval from the McGill University Animal Care Committee. 6–12-week-old mice of both sexes were used for all experiments. Mice were maintained on a 12-h light/dark cycle (light 7am–7pm; dark 7pm–7am), with an ambient temperature of between 20 and 23 °C and between 40 and 60% humidity.

ZIKV^CDN^ (p.8) or ZIKV^BR^ (p.5) were injected intravenously (i.v.) at 1 × 10^5^ PFU or 1 × 10^6^ PFU (ZIKV^CDN^); and 1 × 10^6^ PFU or 5 × 10^6^ PFU (ZIKV^BR^). Mice injected with UV-inactivated ZIKV received a dose of inactivated virus equivalent to 1 × 10^6^ PFU. Mock-infected mice were injected with an identical volume of mock-infected Vero cell culture supernatant (prepared in parallel to ZIKV stocks) i.v. Co-infected mice received 1 × 10^6^ PFU each of ZIKV^CDN^ and ZIKV^BR^. For pI:C and rIFN-β experiments, mice were injected i.p. with either PBS control, 200 μg pI:C (InvivoGen), or 0.5 μg of rIFN-β (Peprotech) on days 2 and 3 post-infection. For BrdU accumulation experiments, mice were given an i.p. injection of 2 mg BrdU (Fisher Scientific) prior to infection, followed by maintenance on 0.8 mg/mL BrdU in their drinking water. For ONX 0914 experiments, mice were treated s.c. with 8 mg/kg of ONX 0914 (Cedarlane), a dose that preferentially targets LMP7^44^, resuspended in a 1:1 mixture of 100% EtOH and PBS, or vehicle control, immediately prior to infection.

### Peptides, MHC class I tetramer, and flow cytometry antibodies

PrM_145__–__153_ (ISFPTTLGM), PrM_169__–__177_ (ATMSYECPM), Env_294__–__302_ (IGVSNRDFV), Env_635__–__645_ (MAVDMQTLTPV), NS1_969__–__978_ (YSLECDPAVI), NS2A_1237__–__1244_ (VSFIFRAN), NS3_1866__–__1874_ (PSVRNGNEI), NS5_2783__–__2792_ (CAEAPNMKII), NS5_2899__–__2906_ (SSWLWKEL) and NS5_2993__–__3000_ (RAIWYMWL) peptides were synthesized by Bio Basic Canada Inc. OVA_257__–__264_ peptide (SIINFEKL) was synthesized by Bio-Synthesis Inc. H-2D^b^ MHC-I tetramer (H-2D^b^ Env_294__–__302_) was provided as PE-conjugated tetramer by the NIH tetramer core facility (used at 1:100 dilution). Fc blocking was performed using TruStain fcX (anti-mouse CD16/CD32, clone 93, BioLegend, catalog #101320, 1:100 dilution). The following antibodies were used in an appropriate combination of fluorochromes: CD3ε (clone 145-2C11, BioLegend, catalog #100306, 1:200 dilution), CD4 (clone GK1.5, BD Biosciences, catalog #563790, 1:400 dilution), CD8α (clone 53–6.7, BioLegend, catalog #100725, 1:400 dilution), CD11a (clone M17/4, BioLegend, catalog #101124 and #101106, 1:300 dilution), CD11c (clone N418, BioLegend, catalog #117328, 1:300 dilution), CD19 (clone 1D3/CD19, BioLegend, catalog #152404, 1:300 dilution), CD49d (clone R1-2, BioLegend, catalog #103608, 1:200 dilution), CD62L (clone MEL-14, BioLegend, catalog #104438, 1:400 dilution), CD80 (clone 16-10A1, BioLegend, catalog #104708, 1:300 dilution), CD86 (clone GL-1, BioLegend, catalog #105014, 1:500 dilution), CD127 (clone A7R34, BioLegend, catalog #135010, 1:100 dilution), Granzyme B (clone GB11, BioLegend, catalog #515406, 1:50 dilution), IFN-γ (clone XMG1.2, BioLegend, catalog #505806, 1:200 dilution), Ki67 (clone 16A8, BioLegend, catalog #652410, 1:100 dilution), KLRG1 (clone 2F1/KLRG1, BioLegend, catalog #138416, 1:100 dilution), MHC-II (clone M5/114.15.2, BioLegend, catalog #116422, 1:500 dilution), NK1.1 (clone PK136, BioLegend, catalog #108706, 1:300 dilution), and the following isotype controls used at the same dilution as the corresponding antibody: IgG1-AlexaFluor647 (clone MOPC-21, BioLegend, catalog #400136) and IgG1-FITC (clone MOPC-21, BioLegend, catalog #400138).

### Flow cytometry staining and ex vivo restimulation

For flow cytometry analysis of cells in the blood, blood was collected, and erythrocytes lysed using Vitalyse (Cedarlane), cells were incubated with TruStain fcX (anti-mouse CD16/CD32, clone 93, BioLegend, catalog #101320, 1:100 dilution) and stained using indicated antibodies, followed by fixation with IC Fixation Buffer (eBioscience). For spleen analysis, spleens were isolated and mechanically disrupted to generate single-cell suspensions. Erythrocytes were lysed with Ammonium-Chloride-Potassium (ACK) buffer (0.15 M NH_4_Cl, 1 mM KHCO_3_, and 0.1 mM Na_2_EDTA in distilled H_2_O, pH 7.2), cells were incubated with TruStain fcX (anti-mouse CD16/CD32, clone 93, BioLegend, catalog #101320, 1:100 dilution) and stained with the indicated antibodies, followed by fixation using IC Fixation Buffer (eBioscience). For tetramer staining, following erythrocyte lysis cells were incubated with TruStain fcX (anti-mouse CD16/CD32, clone 93, BioLegend, catalog #101320, 1:100 dilution) prior to staining for 45 min with H-2D^b^ Env_294__–__302_ tetramer, with gentle vortexing every 20 min, followed by surface antibody staining and fixation with IC Fixation Buffer (eBioscience). For dendritic cell detection, spleens were cut into pieces and incubated with 10 ng/mL DNase and 1 mg/mL collagenase for 30 min at 37 °C and 5% CO_2_ prior to generation of a single-cell suspension by mechanical disruption through a 70-micron basket filter (Fisher Scientific), erythrocyte lysis and antibody staining. Intracellular staining for granzyme B was performed using Perm/Wash Buffer (eBioscience) following fixation with IC Fixation Buffer (eBioscience). Intracellular staining for Ki67 was performed using the FoxP3/Transcription Factor Staining Buffer Set (eBioscience), as per the manufacturer’s instructions. Detection of BrdU accumulation was performed using the Phase-Flow FITC BrdU Kit (BioLegend), as per the manufacturer’s instructions. Staining for TCR Vβ diversity was performed using the Anti-Mouse TCR Vβ Screening Panel (BD Biosciences), as per the manufacturer’s instructions. Samples were analyzed with a BD LSRFortessa flow cytometer (BD Biosciences), FACS Diva (BD Biosciences), and FlowJo software (BD Biosciences).

For ex vivo restimulation, spleens were harvested and processed as above. Erythrocytes were lysed with ACK buffer and samples were simulated for 5.5 h at 37 °C with 5% CO_2_ in the presence of 200 nM Env_294__–__302_ peptide or media alone and brefeldin A (BioLegend). Cells were then stained with surface antibodies and fixed with IC Fixation Buffer (eBioscience), followed by intracellular staining for IFN-γ in Perm/Wash Buffer (eBioscience). Samples were analyzed with a BD LSRFortessa flow cytometer (BD Biosciences), FACS Diva (BD Biosciences), and FlowJo software (BD Biosciences).

A representative gating strategy is provided in Supplementary Fig. 10.

### Quantification of IFN-I

To extract serum, blood was collected via cardiac puncture and centrifuged at 4700 × *g* for 10 min at 4 °C. Bioactive IFN-I (both IFN-α and IFN-β) in serum was quantified 12, 24, and 72 hpi and 7 dpi using the B16-blue IFN-α/β reporter cell line according to the manufacturer’s instructions (InvivoGen). Briefly, 75,000 cells were incubated for 24 h at 37 °C and 5% CO_2_ with 20 μL of serum (performed in duplicate) in a 96-well plate (ThermoFisher Scientific). Following incubation, 20 μL of supernatant was transferred to a flat-bottomed 96-well assay plate (Corning) and incubated with 180 μL of QUANTI-Blue (InvivoGen) for 1–5 h. IFN-I protein expression corresponds to the absorption at 655 nm, read on an EnSpire Multimode Plate Reader (PerkinElmer).

### In vitro BMDC generation and OT-I co-culture

Bone marrow was extracted from C57BL/6 WT mice and cultured in the presence of 20 ng/mL granulocyte-macrophage colony-stimulating factor (PeproTech) in “complete DC medium” containing RPMI-1640 (Corning) medium with 10% heat-inactivated FBS (Hyclone), 2 mM l-glutamine (Wisent), 100 U/mL penicillin–streptomycin (Wisent), and 0.01% β-mercaptoethanol (Gibco) at 37 °C with 5% CO_2_. After 8–10 days, DCs were collected and 1 × 10^4^ cells were infected with ZIKV^CDN^, ZIKV^BR^ or incubated with UV-inactivated ZIKV^CDN^ or UV-inactivated ZIKV^BR^ at a MOI of 5 for 6 h at 37 °C with 5% CO_2_. Following this incubation, the infection media was removed and DCs were stimulated with 0.5 ng/mL LPS (Sigma) in the presence of 3 μg whole OVA protein (Worthington Biochemical Corporation) or 4 nM OVA_257__–__264_ peptide (Bio-Synthesis Inc.), or for some experiments, with 3 μg of fluorescein-labeled whole OVA protein (ThermoFisher). Four hours later, cells were either assayed for activation and OVA-Fluorescein uptake, or 1 × 10^5^ e450 Proliferation Dye (eBioscience)-labeled OT-I CD8 T cells were added to the culture. CD8 T cell proliferation was analyzed 3 days later with a BD LSRFortessa flow cytometer (BD Biosciences), FACS Diva software (BD Biosciences), and FlowJo software (BD Biosciences).

For isolation of OT-I CD8 T cells, spleens from OT-I mice were harvested, mechanically disrupted to generate a single-cell suspension, and erythrocytes were lysed using Vitalyse (Cedarlane). OT-I CD8 T cells were enriched via negative selection using the EasySep Mouse CD8^+^ T Cell Isolation Kit (STEMCELL Technologies), as per the manufacturer’s instructions. Following isolation, OT-I CD8 T cells were labeled with e450 Proliferation Dye (eBioscience), as per the manufacturer’s instructions.

### Quantification of viral burden

ZIKV^CDN^-, ZIKV^BR^-, UV-ZIKV- or mock-infected mice were euthanized 12 hpi or 3-, 5-, or 7-dpi, and spleens were collected and weighed. Total RNA was harvested by Trizol extraction according to the manufacturer’s instructions and RNA concentration was determined using a NanoDrop 2000 spectrophotometer. Quantification of ZIKV RNA was determined by TaqMan one-step RT-qPCR on a Bio-Rad CFX96 Touch Real-Time System using an iTaQ Universal Probe One-Step Kit (Bio-Rad) under standard cycling conditions. The primer set used to detect ZIKV RNA (generated by Integrated DNA Technologies) can be found in Supplementary Table 1^22,65,66^. A standard curve was generated of C_t_ value versus log_10_(PFU) using serial 10-fold dilutions of ZIKV RNA extracted from previously titered viral stocks for each ZIKV isolate. To control for RNA content, each reaction contained a standard amount (1 μg) of RNA and quality of the RNA extraction was assessed by 260/280 and 260/230 ratios. Viral burden is expressed as PFU equivalents per gram of tissue after comparison with the standard curve. The LOD was set as the average PFU equivalent per gram of tissue from the mock-infected samples.

For plaque assays, ZIKV^CDN^- and ZIKV^BR^-infected mice were sacrificed 7 dpi and spleens and kidneys were collected into 1 mL ZIKV harvest media (DMEM supplemented with 2% heat-inactivated FBS, 1% l-Glutamine, 1% non-essential amino acids, 1% Penicillin–Streptomycin and 15 mM HEPES). In addition, the brain and ovaries were similarly harvested from ZIKV^BR^-infected mice 7 dpi, and the spleen and kidney were harvested from ZIKV^BR^-infected mice 14 dpi. Organs were weighed prior to homogenization and cellular debris was cleared by centrifugation at 935 × *g* for 10 min. Supernatants were aliquoted and stored at −80 °C until use for plaque assay. Plaque assays were conducted as above. The LOD was set at 1 plaque counted at the lowest dilution plated for plaque assay.

### RT-qPCR analysis

ZIKV^CDN^-, ZIKV^BR^-, or mock-infected mice were euthanized 12, 24, or 72 hpi, and total RNA was extracted as described above and reverse transcribed using the Maxima H Minus cDNA Synthesis Master Mix with dsDNase (Thermo Scientific) according to the manufacturer’s instructions. RT-qPCR was performed in duplicate in 96-well PCR plates (Bio-Rad) using SensiFAST SYBR Lo-ROX mix (Bioline) in a Bio-Rad CFX96 Touch Real-Time System under standard cycling conditions. Data were analyzed using CFX Maestro software (Bio-Rad) and relative mRNA levels were calculated in Excel (Microsoft) using the ΔΔCT method^67^ using TATA-binding protein (*Tbp*) expression as an internal control, and plotted as fold change by normalizing to mock-infected samples. Primers used to detect *Ifitm3*, *Ifna*, *Ifnb1*, *Ifng*, *Irf7*, *Isg15*, *Oas1a*, *Psmb8,* and *Tbp* mRNA (generated by Integrated DNA Technologies) can be found in Supplementary Table 1.

### Statistical analyses

Data were analyzed using GraphPad Prism 9 software. Specific tests for determining statistical significance are indicated in the figure legends. *p-*values of <0.05 were considered statistically significant.

### Reporting summary

Further information on experimental design is available in the Nature Research Reporting Summary linked to this paper.

## Supplementary information

Supplementary Information

Reporting summary

## Data Availability

The authors declare that the main data supporting the findings of this study are available within the article and its Supplementary Information files. Source data are provided with this paper.

## Source data

Source Data
