# Supplementary material for: An epidemic Zika virus isolate suppresses antiviral immunity by disrupting antigen presentation pathways
Source: Nat Commun. 2021 Jun 30;12:4051. doi: 10.1038/s41467-021-24340-0 (PMC8245533; doi:10.1038/s41467-021-24340-0)
Supplement: Supplementary file 1 — Supplementary Information [file 41467_2021_24340_MOESM1_ESM.pdf]

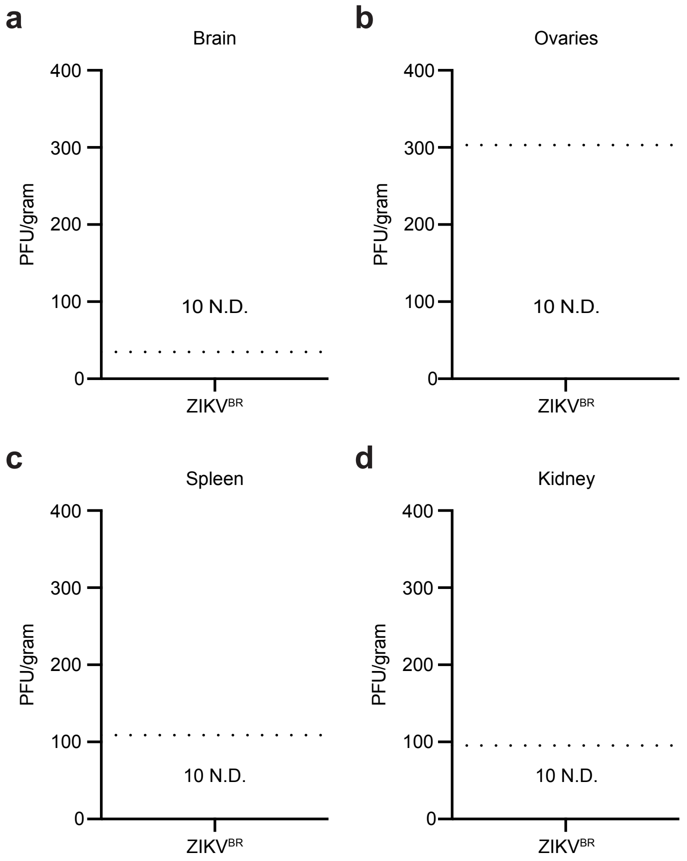

Supplementary Figure 1. ZIKV^{BR} is not detected in brain or ovaries 7 dpi and is cleared 14 dpi. **a-b** Viral burden in the brain (**a**) and ovaries (**b**) was quantified via plaque assay 7 dpi with ZIKV^{BR}. **c-d** Viral burden in the spleen (**c**) and kidney (**d**) was quantified via plaque assay 14 dpi with ZIKV^{BR}. N.D. indicates no data recorded above the LOD (dotted line). Data are pooled from two experiments with n=5 mice per group.Source data are provided as a Source Data file.

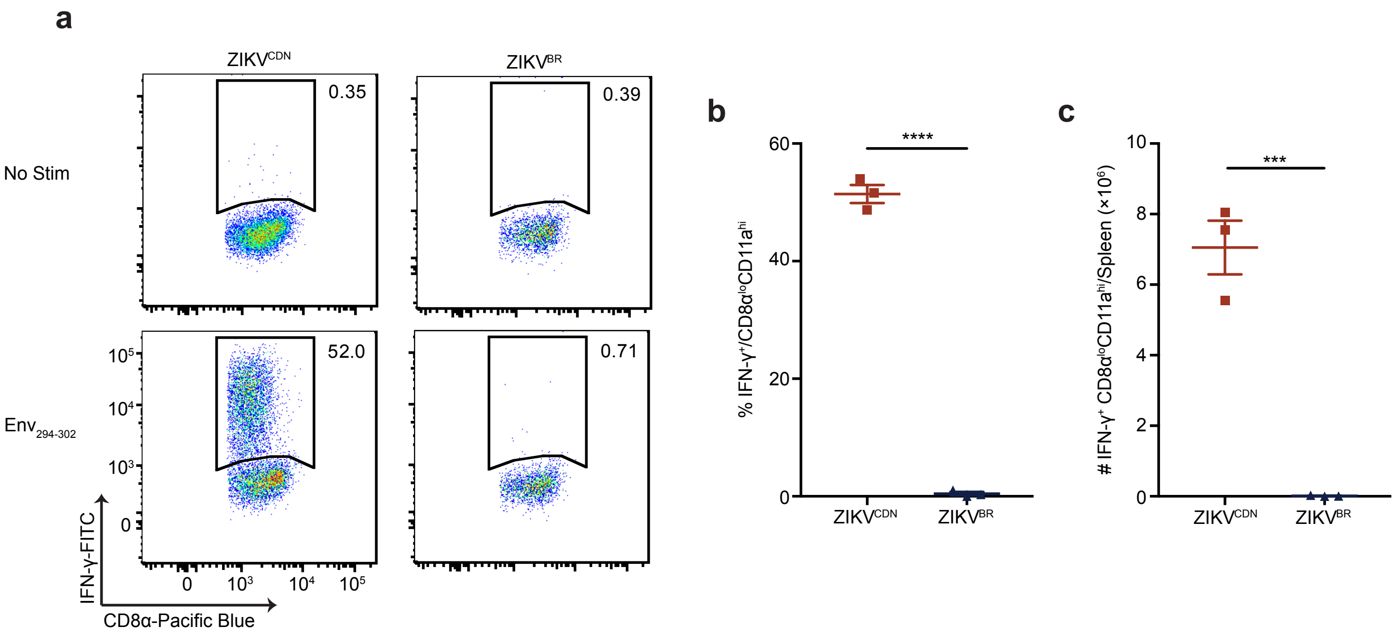

Supplementary Figure 2. ZIKV^{BR} does not induce a CD8 T cell response against the Env$_{294\text{-}302}$ epitope.

a-c Representative flow cytometry plots of IFN-γ production by CD8α^{lo}CD11a^{hi} CD8 T cells from ZIKV^{CDN}- and ZIKV^{BR}-infected mice following incubation for 5.5 hours with media alone (top) or Env$_{294\text{-}302}$ peptide (bottom) in the presence of brefeldin A (**a**). Frequency (**b**) and number (**c**) of IFN-γ^{+} CD8α^{lo}CD11a^{hi} CD8 T cells in the spleen 7 dpi with ZIKV^{CDN} or ZIKV^{BR}, after restimulation for 5.5 hours with media alone or Env$_{294\text{-}302}$ peptide in the presence of brefeldin A. Data are representative of two independent experiments with n=3 mice per group, and are shown as mean ± SEM. Data were analyzed with a two-tailed, unpaired Student's t-test. In **b**, p = 0.000005 and in **c**, p = 0.0008. *** $p < 0.001$ **** $p < 0.0001$. Source data are provided as a Source Data file.

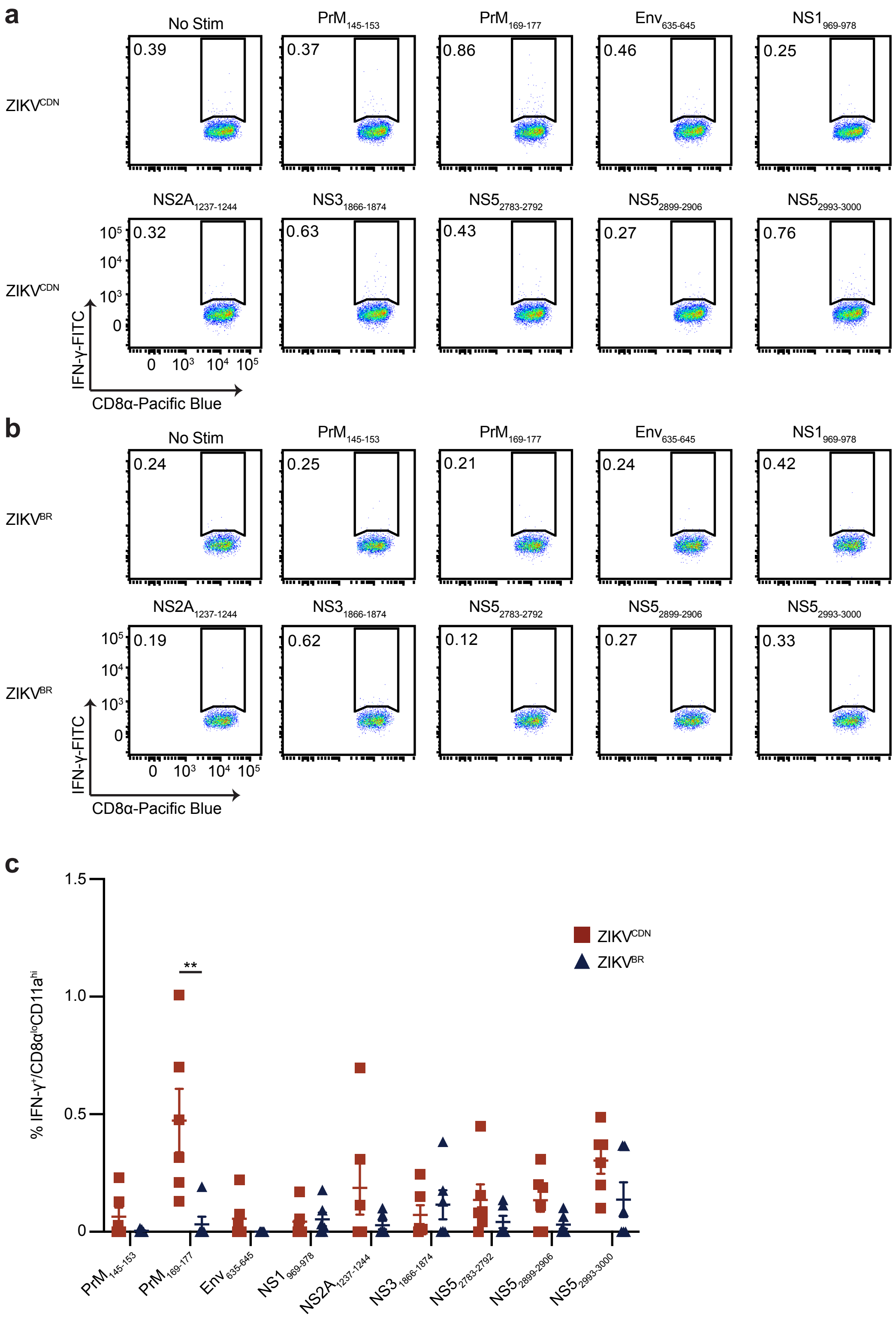

Supplementary Figure 3. CD8 T cell response to ZIKV-derived peptides.

a-c Representative flow cytometry plots of IFN-γ production by CD8α^{lo}CD11a^{hi} CD8 T cells from ZIKV^{CDN}- (**a**) and ZIKV^{BR}-infected (**b**) mice and frequency (**c**) of IFN-γ^{+} CD8α^{lo}CD11a^{hi} CD8 T cells in the spleen 7 dpi with ZIKV^{CDN} or ZIKV^{BR} following incubation for 5.5 hours with media alone (No Stim) or indicated ZIKV-derived peptides in the presence of brefeldin A. Data are pooled from two independent experiments with n=3 mice per group, and are shown as mean ± SEM. Data for each peptide were analyzed by two-tailed, unpaired Student's t-test. PrM$_{169-177}$ $p = 0.0098$. ** $p < 0.01$. Source data are provided as Source Data file.

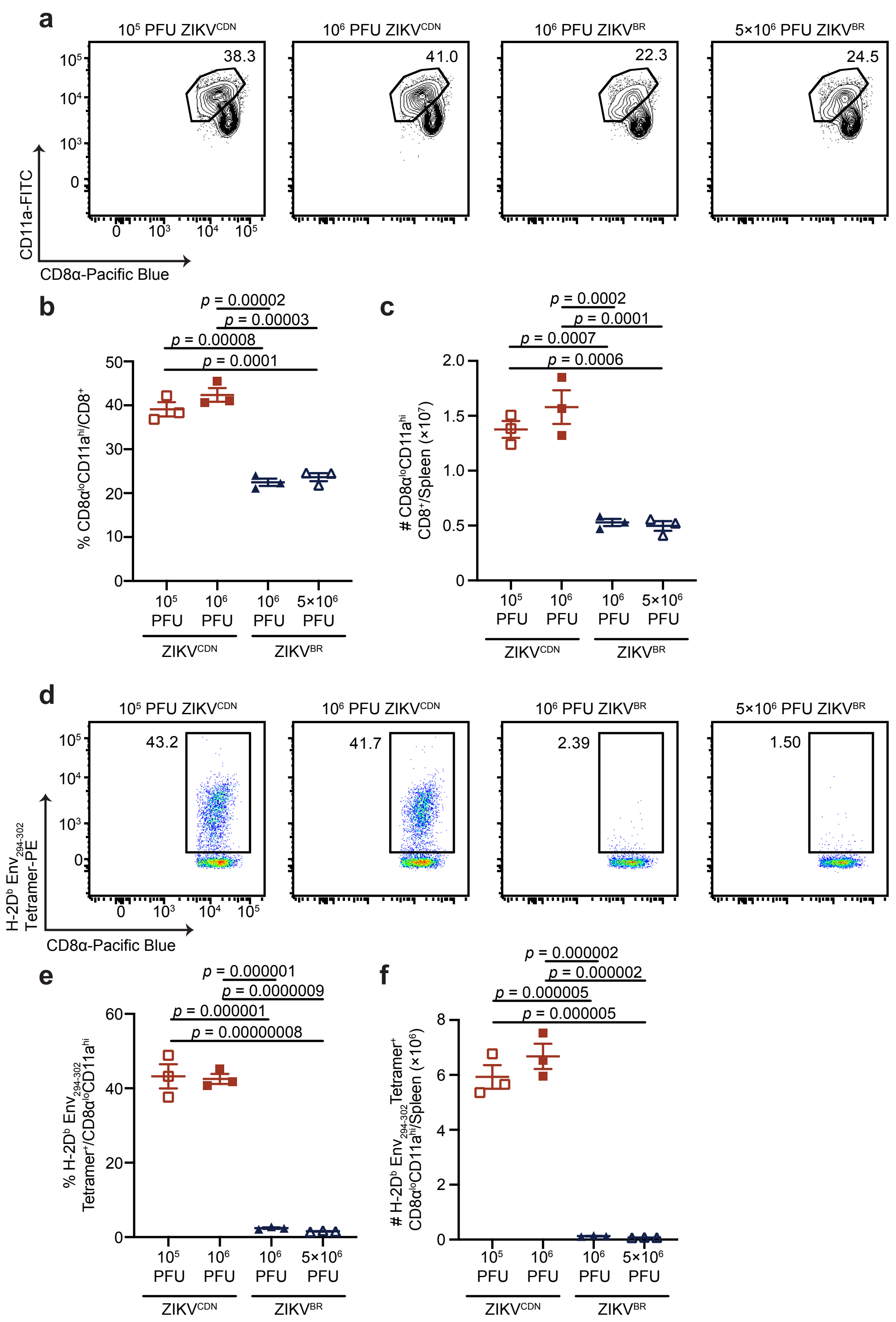

Supplementary Figure 4. Infection dose does not impact the CD8 T cell response to ZIKV^{CDN} or ZIKV^{BR}.

a-c Representative flow cytometry plots (**a**), frequency (**b**) and number (**c**) of CD8α^{lo}CD11a^{hi} CD8 T cells from mice 7 dpi with 10^5 PFU ZIKV^{CDN}, 10^6 PFU ZIKV^{CDN}, 10^6 PFU ZIKV^{BR}, or 5×10^6 PFU ZIKV^{BR}.

d-f Representative flow cytometry plots (**d**), frequency (**e**) and number (**f**) of H-2D^b Env$_{294\text{-}302}$ tetramer-positive CD8α^{lo}CD11a^{hi} CD8 T cells from mice 7 dpi with 10^5 PFU ZIKV^{CDN}, 10^6 PFU ZIKV^{CDN}, 10^6 PFU ZIKV^{BR}, or 5×10^6 PFU ZIKV^{BR}. Data are representative of three independent experiments with n=3 mice per group, and are shown as mean ± SEM. Data were analyzed by one-way ANOVA with Tukey's post-test of multiple comparisons. Source data are provided as Source Data file.

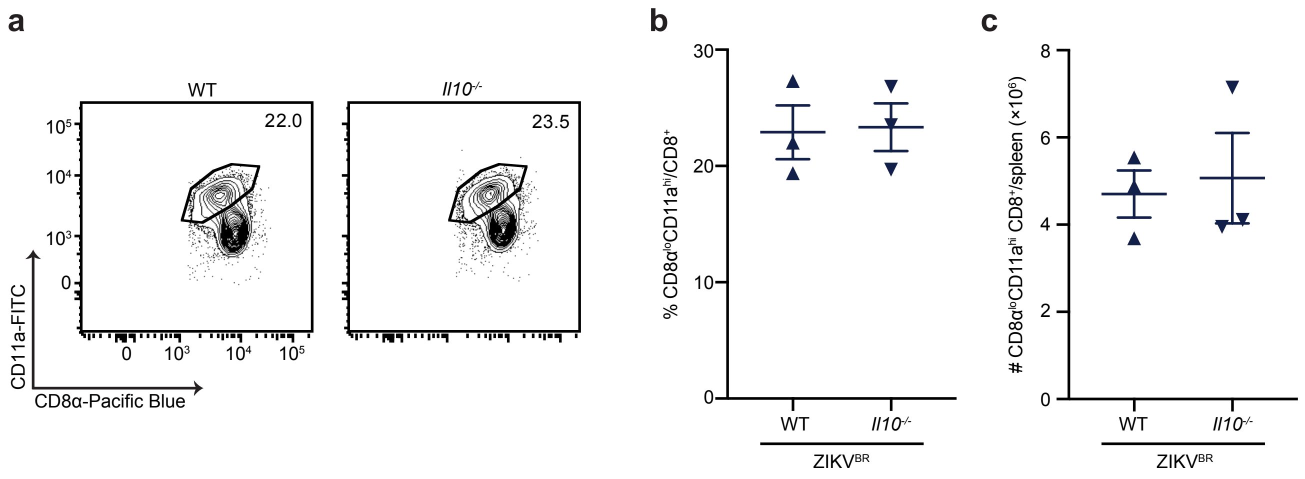

Supplementary Figure 5. IL-10 does not limit the CD8 T cell response to $ZIKV^{BR}$ infection.
a-c Representative flow cytometry plots of $CD8\alpha^{lo}CD11a^{hi}$ CD8 T cells from wild-type (WT) and *Il10*$^{-/-}$ mice 7 dpi with $ZIKV^{BR}$ (**a**). Frequency (**b**) and number (**c**) of $CD8\alpha^{lo}CD11a^{hi}$ CD8 T cells in the spleen from WT and *Il10*$^{-/-}$ mice 7 dpi with $ZIKV^{BR}$. Data are representative of two independent experiments with n=3 mice per group, and are shown as mean ± SEM. Data were analyzed with a two-tailed, unpaired Student's t-test. Source data are provided as a Source Data file.

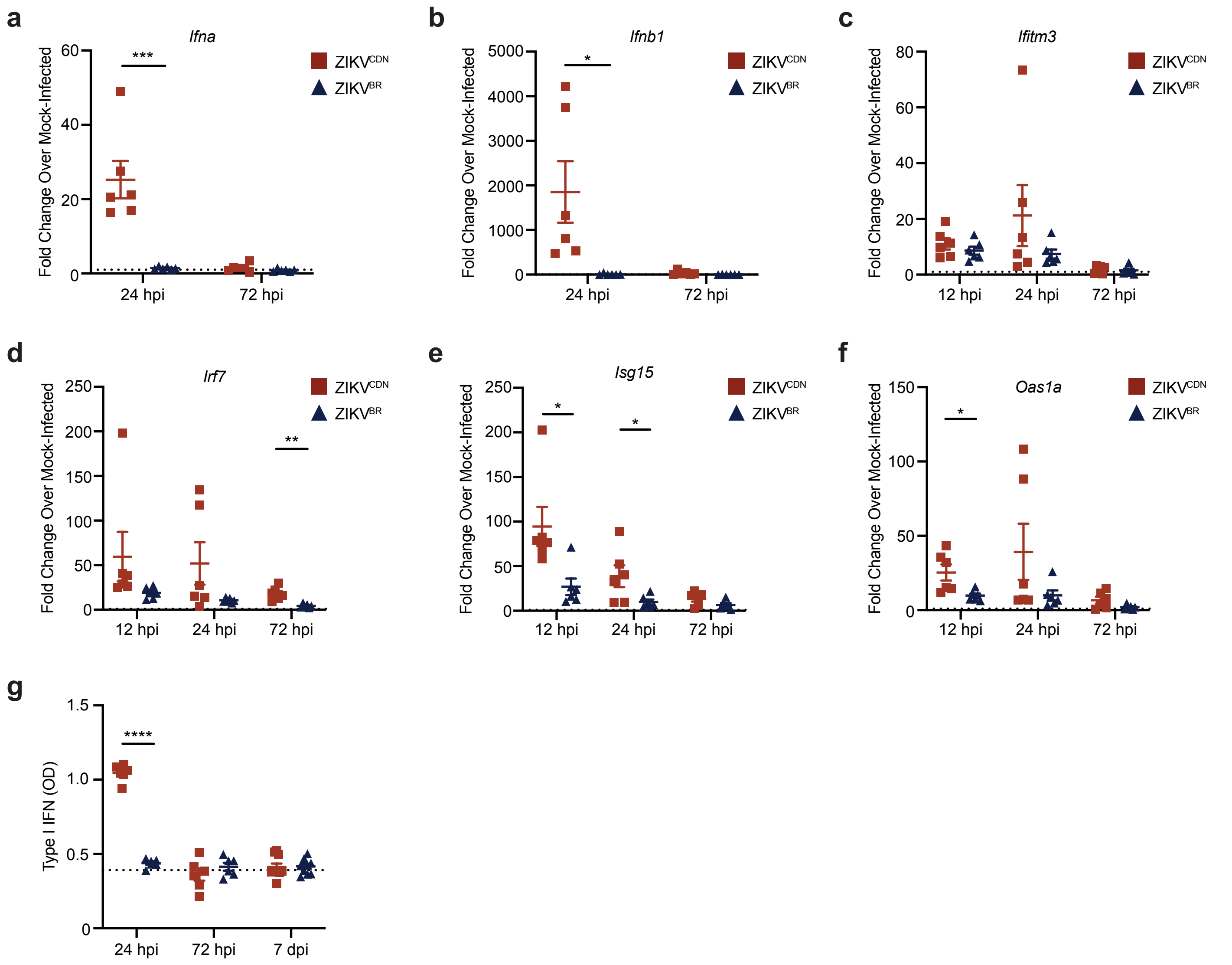

Supplementary Figure 6. Expression of type I IFN and IFN-stimulated gene transcripts over time.

a-f *Ifna* (**a**) and *Ifnb1* (**b**) mRNA expression in the spleen were analyzed by RT-qPCR 24 and 72 hpi with $ZIKV^{CDN}$ or $ZIKV^{BR}$, or mock infection. *Ifitm3* (**c**), *Irf7* (**d**), *Isg15* (**e**) and *Oas1a* (**f**) mRNA expression in the spleen were analyzed by RT-qPCR 12, 24 and 72 hpi with $ZIKV^{CDN}$ or $ZIKV^{BR}$, or mock infection. Data are expressed as fold change over expression in mock-infected mice at the respective timepoints. Dotted line indicates fold change of 1. In **a** $p = 0.0008$, in **b** $p = 0.0233$, in **d** $p = 0.0013$, in **e** 12 hpi $p = 0.0175$ and 24 hpi $p = 0.0437$, in **f** $p = 0.0193$. **g** Total bioactive IFN-I were analyzed in the serum 24 and 72 hpi, as well as 7 dpi with $ZIKV^{CDN}$ or $ZIKV^{BR}$ using the B16-Blue reporter cell line. Dotted line indicates average OD in the serum of mice 24 hours post-mock infection. In **g** $p = 5\times10^{-10}$. In **a**-**f**, data are pooled from two independent experiments with n=3 mice per group. In **g**, data are pooled from two independent experiments with n=3 mice per group (24 hpi and 72 hpi) or n=5 mice per group (7 dpi). All data are shown as mean ± SEM. Data were analyzed with a two-tailed, unpaired Student's t-test at each time point. * $p < 0.05$, ** $p < 0.01$, *** $p < 0.001$, and **** $p < 0.0001$. Source data are provided as a Source Data file.

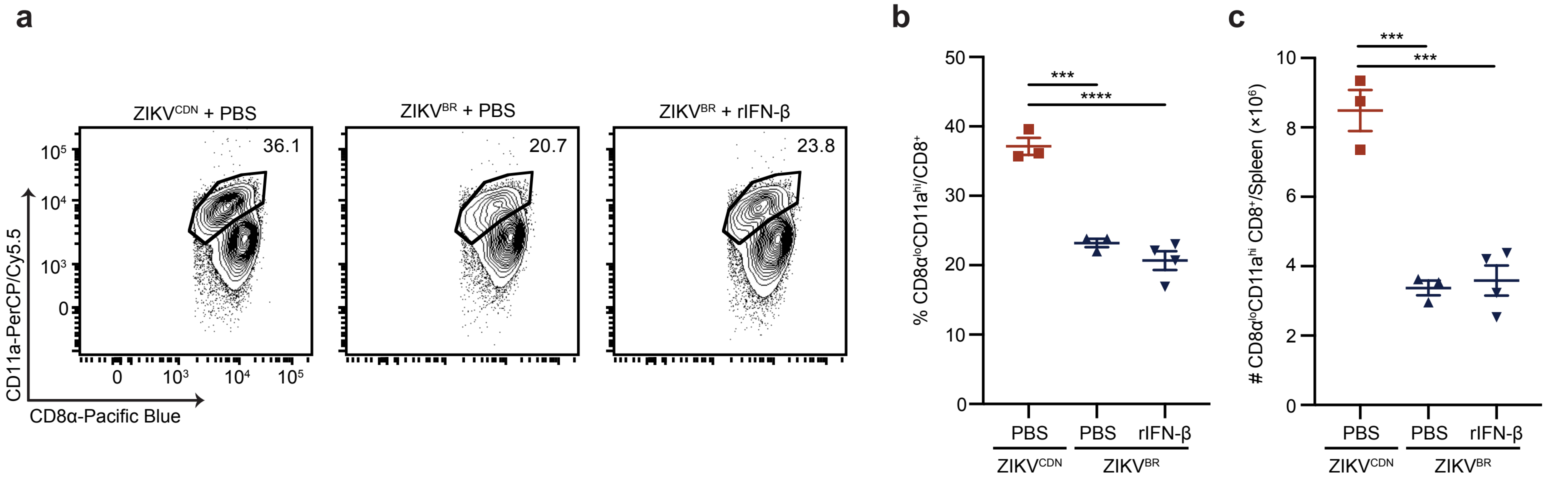

Supplementary Figure 7. IFN-β treatment does not restore the CD8 T cell response to ZIKV^{BR} infection.

a-c Representative flow cytometry plots of CD8α^{lo}CD11a^{hi} CD8 T cells from ZIKV^{CDN}- or ZIKV^{BR}-infected mice treated with PBS or ZIKV^{BR}-infected mice treated with murine rIFN-β, 2 and 3 dpi (**a**). Frequency (**b**) and number (**c**) of CD8α^{lo}CD11a^{hi} CD8 T cells in the spleen 7 dpi with ZIKV^{CDN} or ZIKV^{BR} after treatment with either PBS or murine rIFN-β 2 and 3 dpi. Data are representative of two independent experiments with n=3 (ZIKV^{CDN}+PBS and ZIKV^{BR}+PBS) or 4 (ZIKV^{BR}+rIFN-β) mice per group, and are shown as mean ± SEM. Data were analyzed by one-way ANOVA with Tukey's post-test of multiple comparisons. In **b**, ZIKV^{CDN}+PBS versus ZIKV^{BR}+PBS $p = 0.0003$ and ZIKV^{CDN}+PBS versus ZIKV^{BR}+rIFN-β $p = 0.000058$. In **c**, ZIKV^{CDN}+PBS versus ZIKV^{BR}+PBS $p = 0.0003$ and ZIKV^{CDN}+PBS versus ZIKV^{BR}+rIFN-β $p = 0.0003$. *** $p < 0.001$, **** $p < 0.0001$. Source data are provided as a Source Data file.

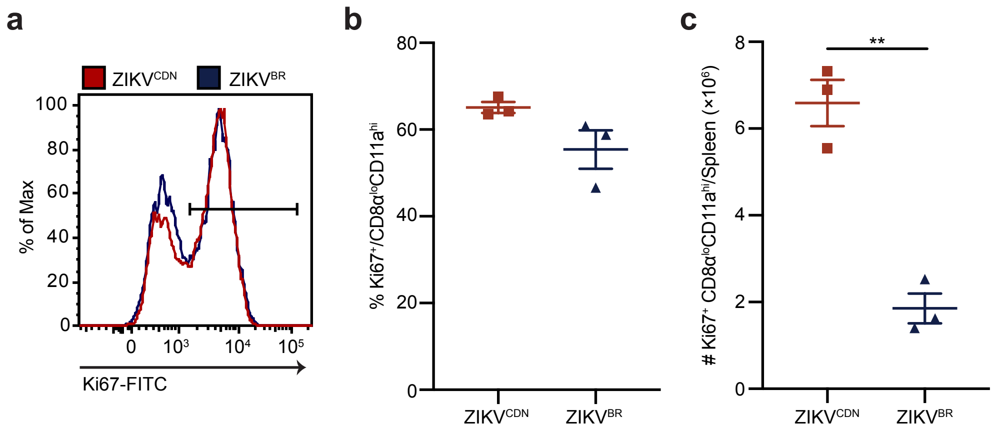

Supplementary Figure 8. Antigen-experienced CD8 T cells enter cell cycle equivalently during ZIKV^{CDN} and ZIKV^{BR} infection.

a-c Representative histogram (**a**), frequency (**b**) and number (**c**) of Ki67^{+} CD8α^{lo}CD11a^{hi} CD8 T cells in the spleen 7 dpi with ZIKV^{CDN} or ZIKV^{BR}. Line on histogram indicates gating strategy used to identify Ki67^{+} cells. Data are representative of two independent experiments with n=3 mice per group, and are shown as mean ± SEM. Data were analyzed with a two-tailed, unpaired Student's t-test. In **c**, p = 0.0061. ** p< 0.01. Source data are provided as a Source Data file.

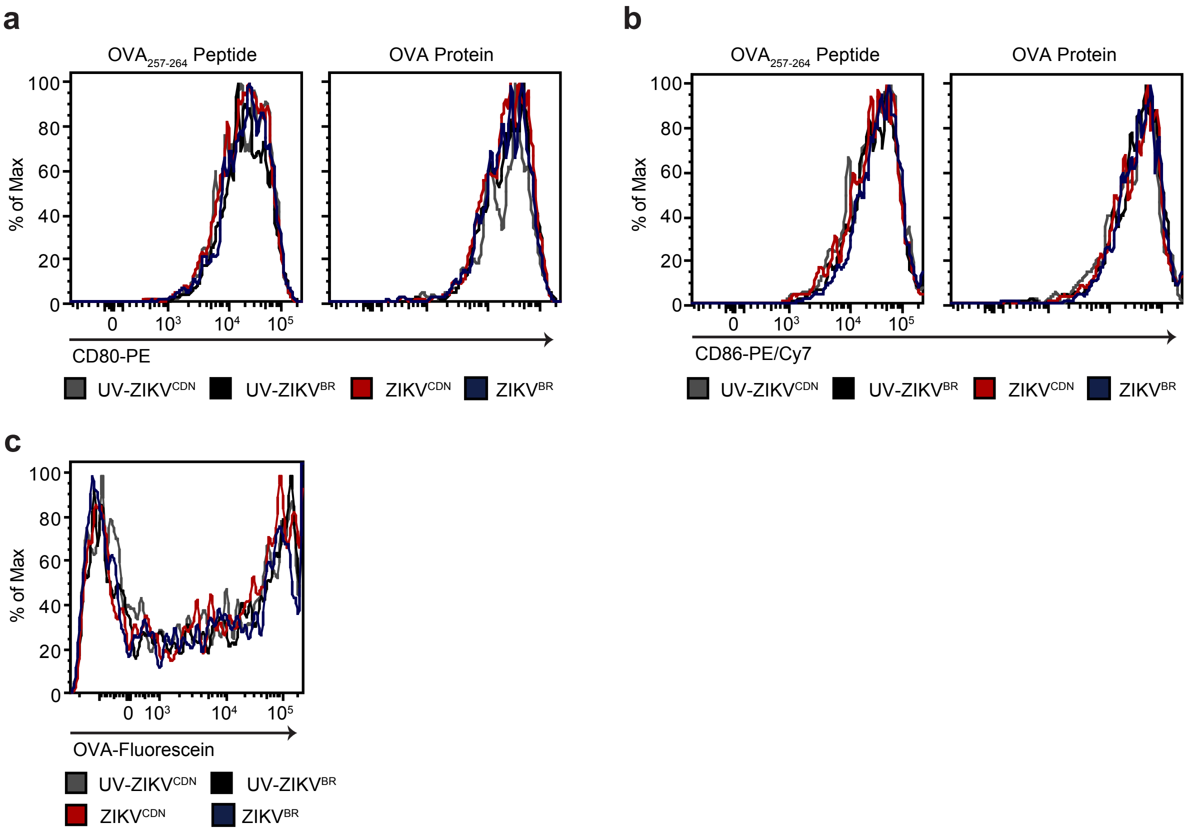

Supplementary Figure 9. ZIKV^{BR} infection does not impact BMDC responsiveness to LPS or protein uptake.

a-c Representative histograms of CD80 (**a**) and CD86 (**b**) expression, and intracellular fluorescein-labeled OVA protein (**c**), gated on CD11c^{+} bone marrow-derived dendritic cells (BMDCs). BMDCs were infected with ZIKV^{CDN} or ZIKV^{BR}, or UV-inactivated ZIKV^{CDN} or ZIKV^{BR}, at a MOI of 5 for 6 hours, followed by a 4-hour incubation with LPS and either OVA$_{257\text{-}264}$ peptide or OVA protein (**a** and **b**), or fluorescein-labeled OVA protein (**c**). Data are representative of two independent experiments.

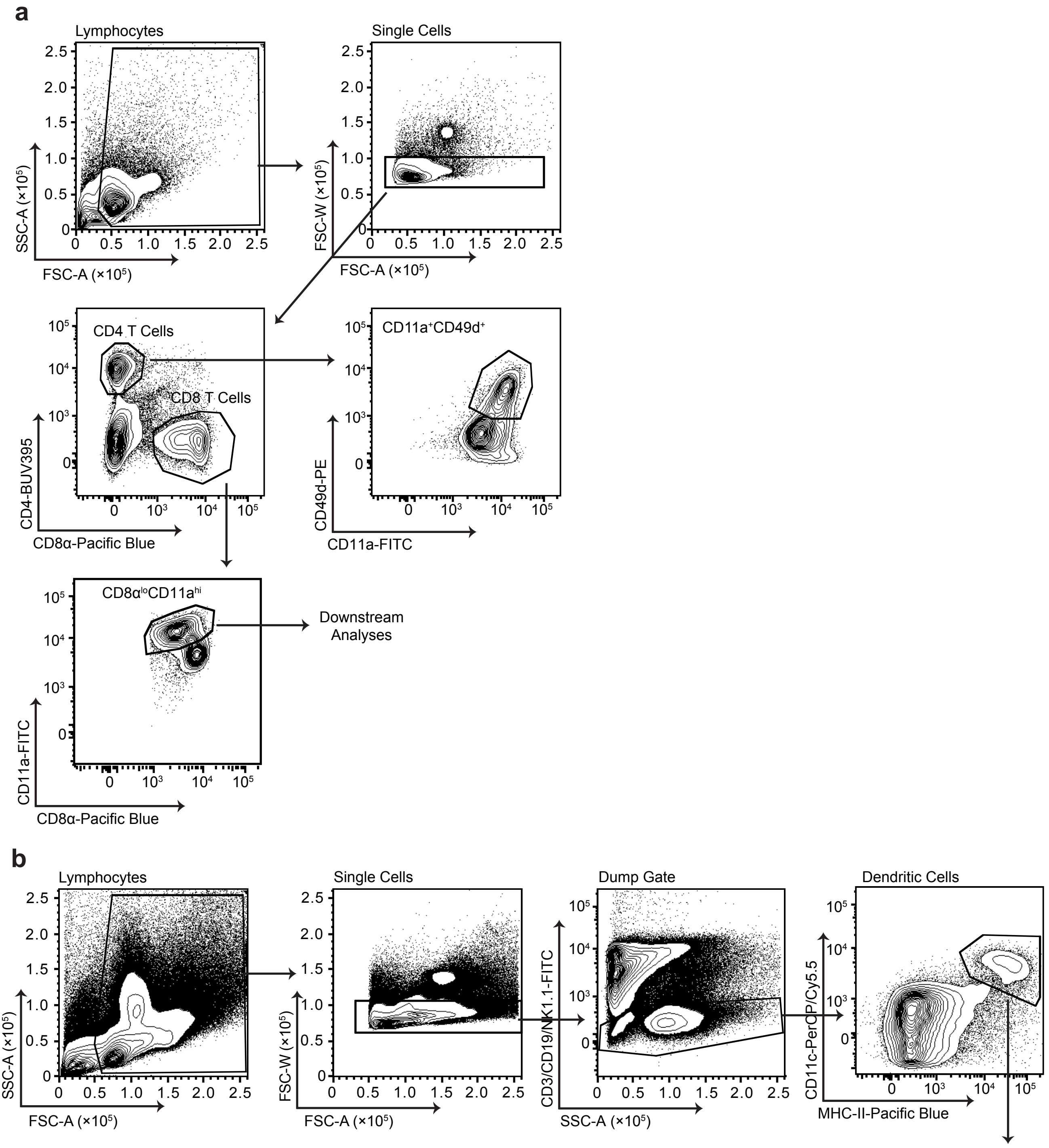

Supplementary Figure 10. Representative gating strategies.

a-b Representative gating strategies to identify CD11a^{+}CD49d^{+} CD4 T cells and CD8α^{lo}CD11a^{hi} CD8 T cells (**a**) and CD3^{-}CD19^{-}NK1.1^{-} MHC-II^{+}CD11c^{+} dendtritic cells (**b**). Representative gating or histograms for downstream analyses are provided in corresponding figures.

Supplementary Table 1. Primers used for RT-qPCR analyses.

Target Gene	Sequence	Orientation
Ifitm3	5’-CTC CGT GAA GTC TAG GGA TCG-3’	Forward
Ifitm3	5’-CAG GAC CGG AAG TCG GAA TC-3’	Reverse
Ifna	5’-TGT CTG ATG CAG CAG GTG G-3’	Forward
Ifna	5’-AAG ACA GGG CTC TCC AGA C-3’	Reverse
Ifnb1	5’-CGT GGG AGA TGT CCT CAA CT-3’	Forward
Ifnb1	5’-CTG AAG ATC TCT GCT CGG ACC-3’	Reverse
Ifng	5’-GCC ATC GGC TGA CCT AGA GA-3’	Forward
Ifng	5’-TCA CCA TCC TTT TGC CAG TTC C-3’	Reverse
Irf7	5’-AGC TTG GAT CTA CTG TGG GC-3’	Forward
Irf7	5’-GGG TTC CTC GTA AAC ACG GT-3’	Reverse
Isg15	5’-CAG CAA TGG CCT GGG ACC TAA-3’	Forward
Isg15	5’-AGG GTA AGA CCG TCC TGG AG-3’	Reverse
Oas1a	5’-CAG CCT TTG ATG TCC TGG GT-3’	Forward
Oas1a	5’-CCC AGC TTC TCC TTA CAC AGT-3’	Reverse
Psmb8	5’-GTG GCT GGG ACA AGA AGG GAC CA-3’	Forward
Psmb8	5’ TAG CTC TGC GGC CAA GGT CGT-3’	Reverse
Tbp	5’-TGG AAT TGT ACC GCA GCT TCA-3’	Forward
Tbp	5’-ACT GCA AAT CGC TTG GG-3’	Reverse
ZIKV	5’-CCG CTG CCC AAC ACA AG-3’	Forward
ZIKV	5’-CCA CTA ACG TTC TTT TGC AGA CAT-3’	Reverse
ZIKV	5’-/56-FAM/AGC CTA CCT/ZEN/ TGA CAA GCA ATC AGA CAC TCA A/3IABkFQ/-3’	Probe
