# Supplementary material for: An epidemic Zika virus isolate suppresses antiviral immunity by disrupting antigen presentation pathways
Source: Nat Commun. 2021 Jun 30;12:4051. doi: 10.1038/s41467-021-24340-0 (PMC8245533; doi:10.1038/s41467-021-24340-0)
Supplement: Supplementary file 2 — Reporting summary [file 41467_2021_24340_MOESM2_ESM.pdf]

nature research

Corresponding author(s): Martin J. Richer

Last updated by author(s): May 6, 2021

Reporting Summary

Nature Research wishes to improve the reproducibility of the work that we publish. This form provides structure for consistency and transparency in reporting. For further information on Nature Research policies, see Authors & Referees and the Editorial Policy Checklist .

Statistics

For all statistical analyses, confirm that the following items are present in the figure legend, table legend, main text, or Methods section.

n/a	Confirmed	
☐	☒	The exact sample size (*n*) for each experimental group/condition, given as a discrete number and unit of measurement
☐	☒	A statement on whether measurements were taken from distinct samples or whether the same sample was measured repeatedly
☐	☒	The statistical test(s) used AND whether they are one- or two-sided<br>*Only common tests should be described solely by name; describe more complex techniques in the Methods section.*
☐	☒	A description of all covariates tested
☐	☒	A description of any assumptions or corrections, such as tests of normality and adjustment for multiple comparisons
☐	☒	A full description of the statistical parameters including central tendency (e.g. means) or other basic estimates (e.g. regression coefficient) AND variation (e.g. standard deviation) or associated estimates of uncertainty (e.g. confidence intervals)
☐	☒	For null hypothesis testing, the test statistic (e.g. *F*, *t*, *r*) with confidence intervals, effect sizes, degrees of freedom and *P* value noted<br>*Give P values as exact values whenever suitable.*
☒	☐	For Bayesian analysis, information on the choice of priors and Markov chain Monte Carlo settings
☒	☐	For hierarchical and complex designs, identification of the appropriate level for tests and full reporting of outcomes
☒	☐	Estimates of effect sizes (e.g. Cohen's *d*, Pearson's *r*), indicating how they were calculated

Our web collection on statistics for biologists contains articles on many of the points above.

Software and code

Policy information about availability of computer code

Data collection	Flow cytometry data were collected using FACSDiva Software (version 8.0, BD Biosciences).
Data analysis	Flow cytometry data were analyzed using Flowjo software (version 9.9.5, BD Biosciences). RT-qPCR data were first analzyed using CFX Maestro software (version 4.1.2434.0124, BioRad) and fold changes or PFU equivalents were calculated using Excel (version 16.43, Microsoft). Type I IFN quantification data were analyzed using Excel. All graphs were made and statistics calculated using Prism 9 (version 9.1.0, GraphPad).

For manuscripts utilizing custom algorithms or software that are central to the research but not yet described in published literature, software must be made available to editors/reviewers. We strongly encourage code deposition in a community repository (e.g. GitHub). See the Nature Research guidelines for submitting code & software for further information.

Data

Policy information about availability of data

All manuscripts must include a data availability statement. This statement should provide the following information, where applicable:
- Accession codes, unique identifiers, or web links for publicly available datasets
- A list of figures that have associated raw data
- A description of any restrictions on data availability

The authors declare that the main data supporting the findings of this study are available within the article and its Supplementary Information files.

Field-specific reporting

Please select the one below that is the best fit for your research. If you are not sure, read the appropriate sections before making your selection.

Life sciences study design

All studies must disclose on these points even when the disclosure is negative.

Sample size	Experiments were conducted with at least 3-5 mice per group, chosen to ensure that data were reproducible and held biological significance. Further, these sample sizes are standard for the field and have been previously used to demonstrate differences following ZIKV infection (PMID 28081442; PMID 28835502; PMID 30677097; PMID 28231312).
Data exclusions	No data were excluded from the analyses.
Replication	The findings herein were reliably reproduced and number of times experiments were repeated are indicated in figure legends (minimum twice for in vivo experiments, range 2-4, minimum 3 times for in vitro experiments).
Randomization	Mice were randomly assigned to indicated groups. For in vitro experiments, different groups were derived from the same cell suspensions/ stock materials so randomization was not required but controlled for.
Blinding	No blinding was performed. All data analysis was undertaken impartially and in a strict unbiased manner and is reported as it was collected. Flow cytometry, RT-qPCR, and B16-Blue assay were collected using automated software that did not require intervention by the experimenters.

Reporting for specific materials, systems and methods

We require information from authors about some types of materials, experimental systems and methods used in many studies. Here, indicate whether each material, system or method listed is relevant to your study. If you are not sure if a list item applies to your research, read the appropriate section before selecting a response.

Materials & experimental systems

n/a	Involved in the study	
☐	☒	Antibodies
☐	☒	Eukaryotic cell lines
☒	☐	Palaeontology
☐	☒	Animals and other organisms
☒	☐	Human research participants
☒	☐	Clinical data

Methods

n/a	Involved in the study	
☒	☐	ChIP-seq
☐	☒	Flow cytometry
☒	☐	MRI-based neuroimaging

Antibodies

Antibodies used	Fc blocking was performed using TruStain fcX (anti-mouse CD16/CD32, clone 93, BioLegend, catalog #101320, 1:100 dilution). The following antibodies were used in an appropriate combination of fluorochromes: CD3ε (clone 145-2C11, BioLegend, catalog #100306, 1:200 dilution), CD4 (clone GK1.5, BD Biosciences, catalog #563790, 1:400 dilution), CD8α (clone 53–6.7, BioLegend, catalog #100725, 1:400 dilution), CD11a (clone M17/4, BioLegend, catalog #101124 and #101106, 1:300 dilution), CD11c (clone N418, BioLegend, catalog #117328, 1:300 dilution), CD19 (clone 1D3/CD19, BioLegend, catalog #152404, 1:300 dilution), CD49d (clone R1-2, BioLegend, catalog #103608, 1:200 dilution), CD62L (clone MEL-14, BioLegend, catalog #104438, 1:400 dilution), CD80 (clone 16-10A1, BioLegend, catalog #104708, 1:300 dilution), CD86 (clone GL-1, BioLegend, catalog #105014, 1:500 dilution), CD127 (clone A7R34, BioLegend, catalog #135010, 1:100 dilution), Granzyme B (clone GB11, BioLegend, catalog #515406, 1:50 dilution), IFN-γ (clone XMG1.2, BioLegend, catalog #505806, 1:200 dilution), Ki67 (clone 16A8, BioLegend, catalog #652410, 1:100 dilution), KLRG1 (clone 2F1/KLRG1, BioLegend, catalog #138416, 1:100 dilution), MHC-II (clone M5/114.15.2, BioLegend, catalog #116422, 1:500 dilution), NK1.1 (clone PK136, BioLegend, catalog #108706, 1:300 dilution), and and the following isotype controls used at the same dilution as the corresponding antibody: IgG1-AlexaFluor647 (clone MOPC-21, BioLegend, catalog #400136) and IgG1-FITC (clone MOPC-21, BioLegend, catalog #400138).
Validation	All antibodies are commercially available and were validated by the manufacturers from which they were purchased. For BioLegend antibodies, validation includes (from https://www.biolegend.com/en-us/quality-control): 1) Staining of 1-3 target cell types with either single- or multi-color analysis detailed in the QC specification (including positive and negative controls). The tested cells can be primary cells and/or cell lines known to be positive or negative for the target antigen. 2) Each batch product is validated by QC testing with a series of dilutions to make sure the product is working within expected antibody titer range. 3) Each batch is compared to an internally established "gold standard" to maintain batch-to-batch consistency. 4) When applicable, our products are side-by-side tested with our competitors' products to make sure that BioLegend's products exceed or are at least the same quality. 5) For most tandem dye-conjugated products, color compensation is examined in order to verify tandem integrity.<br>BD Biosciences antibodies are QC tested to ensure species and antigen reactivity, and are routinely tested for the intended purpose (flow cytometry - see https://www.bdbiosciences.com/us/applications/research/t-cell-immunology/th-1-cells/surface-markers/mouse/buv395-rat-anti-mouse-cd4-gk15/p/563790).

Eukaryotic cell lines

Policy information about cell lines

Cell line source(s)	Vero cells: Dr. Steven Varga, University of Iowa (originally purchased from ATCC); B16-Blue cells: Dr. Maziar Divangahi, McGill University (originally purchased from Invivogen)
Authentication	All cell lines used were authenticated by the manufacturers and checked morphologically under the microscope.
Mycoplasma contamination	All cell lines used tested negative for mycoplasma contamination.
Commonly misidentified lines (See ICLAC register)	No commonly misidentified cell lines were used.

Animals and other organisms

Policy information about studies involving animals; ARRIVE guidelines recommended for reporting animal research

Laboratory animals	6-12 week old wild-type, IL-10 knock-out, and OT-I mice on the C57BL/6 background of both sexes were used for this study. Mice were maintained on a 12-hour light/dark cycle (light 7am-7pm; dark 7pm-7am), with an ambient temperature of between 20°C and 23°C and between 40% and 60% humidity.
Wild animals	The study did not involve wild animals.
Field-collected samples	The study did not involve samples collective from the field.
Ethics oversight	All animal procedures were carried out in accordance with the Canadian Council on Animal Care and were approved by the McGill University Animal Care Committee.

Note that full information on the approval of the study protocol must also be provided in the manuscript.

Flow Cytometry

Plots

Confirm that:

- [x] The axis labels state the marker and fluorochrome used (e.g. CD4-FITC).
- [x] The axis scales are clearly visible. Include numbers along axes only for bottom left plot of group (a 'group' is an analysis of identical markers).
- [x] All plots are contour plots with outliers or pseudocolor plots.
- [x] A numerical value for number of cells or percentage (with statistics) is provided.

Methodology

Sample preparation	For flow cytometry analysis of cells in the blood, blood was collected, and erythrocytes lysed using Vitalyse (Cedarlane), cells were incubated with TruStain fcX (anti-mouse CD16/CD32, clone 93, BioLegend) and stained using indicated antibodies, followed by fixation with IC Fixation Buffer (eBioscience). For spleen analysis, spleens were isolated and mechanically disrupted to generate single-cell suspensions. Erythrocytes were lysed with Ammonium-Chloride-Potassium buffer (0.15 M NH4Cl, 1 mM KHCO3, and 0.1mM Na2EDTA in distilled H2O, pH 7.2), cells were incubated with TruStain fcX (anti-mouse CD16/CD32, clone 93, BioLegend) and stained with the indicated antibodies, followed by fixation using IC Fixation Buffer (eBioscience). For tetramer staining, following erythrocyte lysis cells were incubated with TruStain fcX (anti-mouse CD16/CD32, clone 93, BioLegend) prior to staining for 45 minutes with H-2Db Env294-302 tetramer, with gentle vortexing every 20 minutes, followed by surface antibody staining and fixation with IC Fixation Buffer (eBioscience). For dendritic cell detection, spleens were cut into pieces and incubated with 10 ng/mL DNase and 1 mg/mL collagenase for 30 minutes at 37°C and 5% CO2 prior to generation of a single-cell suspension by mechanical disruption through a 70 micron basket filter (Fisher Scientific), erythrocyte lysis and antibody staining. Intracellular staining for granzyme B was performed using Perm/Wash Buffer (eBioscience) following fixation with IC Fixation Buffer (eBioscience). Intracellular staining for Ki67 was performed using the FoxP3/Transcription Factor Staining Buffer Set (eBioscience), as per the manufacturer's instructions. Detection of BrdU accumulation was performed using the Phase-Flow FITC BrdU Kit (BioLegend), as per the manufacturer's instructions. Staining for TCR Vβ diversity was performed using the Anti-Mouse TCR Vβ Screening Panel (BD Biosciences), as per the manufacturer's instructions. Samples were analyzed with a BD LSRFortessa flow cytometer (BD Biosciences) and FlowJo software (BD Biosciences).<br>For ex vivo restimulation, spleens were harvested and processed as above. Erythrocytes were lysed with ACK buffer and samples were simulated for 5.5 hours at 37°C with 5% CO2 in the presence of 200nM Env294-302 peptide or media alone and brefeldin A (BioLegend). Cells were then stained with surface antibodies and fixed with IC Fixation Buffer (eBioscience), followed by intracellular staining for IFN-γ in Perm/Wash Buffer (eBioscience). Samples were analyzed with a BD LSRFortessa flow cytometer (BD Biosciences) and FlowJo software (BD Biosciences).
Instrument	BD LSRFortessa (BD Biosciences)

Software	Data collected using FACS Diva software (BD) and analyzed using Flowjo (BD Biosciences)
Cell population abundance	No cell sorting was used in this study
Gating strategy	Samples were gated first based on FSC-A and SSC-A to exclude debris (based on small size and low granularity - FSC and SSC lower than approximately 25,000). Next, doublets were excluded by gating FSC-A versus FSC-W, the "positive" population was the smaller population, typically cells with an FSC-W lower than 100,000. Cells were subsequently gated based on expression of lineage-specific markers, such as CD4 for CD4 T cells, CD8 for CD8 T cells, or NK1.1 for natural killer cells. See gating strategy in Supplementary Information. Boundaries between positive and negative populations were determined based on the separation of those populations in the FACS plot, isotype or "Fluorescence Minus One" (FMO) controls where appropriate (e.g. for shifts on a histogram), or previously published analyses (e.g. PMID 28231312 and PMID 19933864 for CD11ahiCD8alo CD8 T cells). See Supplementary Figure S10 for overarching gating strategy. Specific representative gating is provided in each figure where appropriate.

☒ Tick this box to confirm that a figure exemplifying the gating strategy is provided in the Supplementary Information.
